# Home range size and foraging niche predict lead exposure variability in terrestrial birds

**DOI:** 10.1007/s10646-026-03123-7

**Published:** 2026-07-02

**Authors:** Max M Gillings, Riccardo Ton, Simon C Griffith

**Affiliations:** 1 School of Natural Sciences, Faculty of Science and Engineering, Macquarie University, Sydney, NSW 2109 Australia; 2 Cavanilles Institute of Biodiversity and Evolutionary Biology, University of Valencia, Valencia, 46980 Spain

**Keywords:** Sentinel species, Movement ecology, Home range, Lead contamination, Wildlife biomonitoring, Exposure variability

## Abstract

Ecological risk assessments estimate chemical exposures in wildlife from contaminant concentrations within their surrounding environment. This approach precludes a detailed understanding of interactions between biota and contaminant sources. It also overlooks how the uneven and irregular use of space by animals influences variability in exposure. Here, we conducted a meta-analysis to examine whether site-specific variability in lead exposure was associated with the home range size and movement-related traits of terrestrial bird species. We constructed a series of linear models to test the effect of ecological and environmental predictors on variability in lead exposure, which we quantified using the coefficient of variation for site-specific feather lead concentrations. Accounting for variance across study, site, species, and phylogeny, we show that birds with larger home ranges exhibited more variable levels of lead exposure than those with more restricted movement patterns. This relationship was moderated by foraging niche and was significant in ground foraging birds but non-significant in generalists and arboreal-aerial foragers, which may relate to differences in dietary lead sources and niche complexity. Site-specific variability in feather lead concentrations was further structured by feather type, with contour feathers exhibiting lower variability than wing, tail, or mixed feather samples. Our findings highlight how the use of space at varying scales might influence divergent patterns of chemical exposure in terrestrial birds. We discuss how incorporating movement-related traits into wildlife risk assessments can reduce uncertainties associated with spatial variability in exposure and improve the ecological relevance of monitoring outcomes.

**Graphical abstract:**

**Figure:**
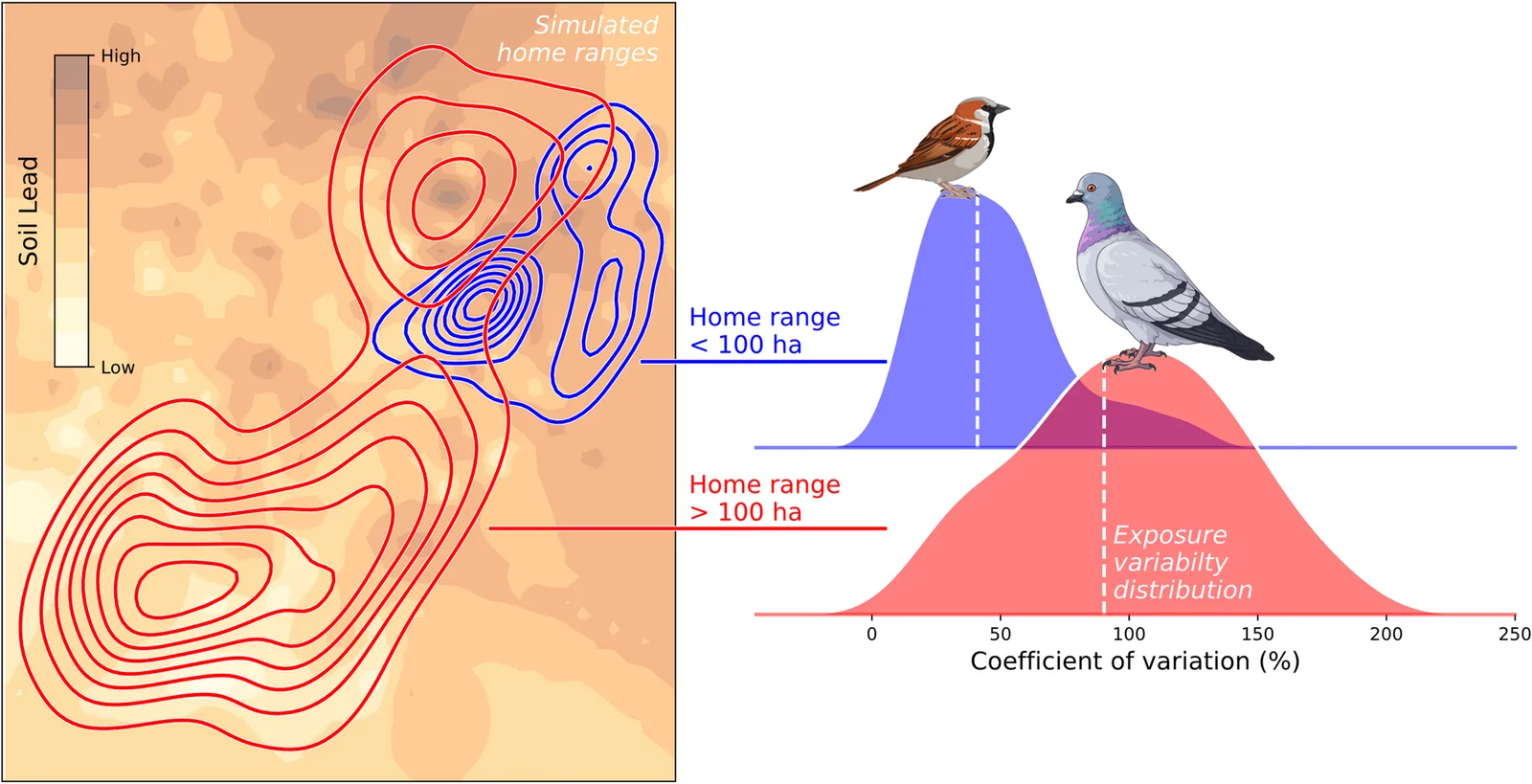

**Supplementary Information:**

The online version contains supplementary material available at https://doi.org/10.1007/s10646-026-03123-7.

## Introduction

Chemical pollution poses a growing yet often underestimated threat to global biodiversity and ecosystem health (Sigmund et al. 2023). Our capacity to monitor and manage chemical threats to wildlife is challenged by the growing complexity of human modified areas (Naidu et al. 2021). Ecological risk assessments circumvent this complexity by estimating exposures in wildlife from average contaminant concentrations in their surrounding environment (Morrissey et al. 2024; US EPA 1996). However, animal movements are not random but show a strong temporal affinity to core areas of quality habitat (Chow et al. 2005). Exposure is therefore rarely constant across an animal’s range, irrespective of its size (Bæk et al. 2023; Hinton et al. 2019). This bears on a common yet untested assumption of research concerning chemical risks to wildlife; that species which are active over large areas are exposed to more spatially variable levels of contamination than those with more restricted patterns of space use (Marinussen and van der Zee 1996). Investigating this assumption is worthwhile because it has implications for the derivation of wildlife exposure data and its wider relevance to the health of other species with similar movement ecologies (Gillings et al. 2024a).

Species traits, especially those related to spatial behaviours, are increasingly recognised for their potential to provide a mechanistic understanding of exposure risk that is transferable across taxa and ecological contexts (Van den Brink et al. 2011). Movement-related traits constrain interactions between biota and the external environment and influence species-specific vulnerabilities to chemical pollutants (Bro et al. 2015; Leeb et al. 2020; Lenhardt et al. 2015; Mayer et al. 2020). For example, interspecific variation in diet and foraging niche drive divergent patterns of exposure in habitats where contaminants are unevenly distributed through food sources and substrates (Durkalec et al. 2022; Gillings et al. 2024b). Migratory behaviours increase the probability of encountering contaminant sources distributed over large geographic areas while simultaneously limiting the duration of contact with those sources (Knutsen and Varian-Ramos 2020; Moore et al. 2018). Home range — a common pattern of space use essential for an animal’s survival and reproduction — delineates the space in which a species is exposed to contaminants in its environment (Bæk et al. 2023; Hinton et al. 2019; Tartu et al. 2018). Thus, behavioural traits such as migration and home range play a key role in shaping spatial and temporal patterns of exposure. However, they also introduce a significant yet often unresolved source of variability in measured and estimated contaminant concentrations in wildlife which warrants further research.

Here, we explore associations between movement-related traits and variability in chemical exposure by systematically reviewing site-specific assessments of lead (Pb) in the feathers of terrestrial bird species with documented home ranges. Birds are commonly used as biomonitors or sentinels of environmental contamination (Pollack et al. 2017) and their movements are frequently tracked to understand habitat use and migratory behaviours (Baak et al. 2024). However, outside of migration, research linking avian movements to exposure remains limited by a lack of spatially explicit data (Morrissey et al. 2024). In the absence of animal tracking data, we use home range size and other movement-related traits to examine correlates of variability in the intensity, frequency and duration with which individuals inhabiting the same environment are exposed to nearby sources of lead contamination.

Also evaluated is the influence of other functional traits (e.g., body mass, trophic level), as well as environmental factors (e.g., urbanisation, contamination source) and methodological considerations (e.g., feather type), that might influence variability in the reviewed feather lead concentrations. For example, body mass correlates with home range size and metabolic rate (Dial et al. 2008), and can influence individual and interspecific patterns of contaminant exposure and uptake and their accumulation in different biological compartments (Buekers et al. 2009; Kuo et al. 2022). Emission sources, such as urban traffic or mining operations, influence the spatial extent and environmental distribution of contaminants like lead and their impacts on exposed wildlife (Gillings et al. 2024b; Grunst et al. 2020). These, along with other methodological factors, are expected to have an additive effect on variability in feather lead concentrations.

We focus on lead because it is a persistent and globally ubiquitous pollutant with a diverse range of anthropogenic sources and exhibits a high degree of environmental heterogeneity (Naidu et al. 2021). It has been widely studied in birds due to its negative effects on survival and reproduction and the associated threat it poses to vulnerable species (Pain et al. 2019). In this study, we consider populations inhabiting contaminated sites where spatial variability in lead exposure is expected to be high. Measurements from feathers are considered as they can be sampled non-destructively, contain readily detectable concentrations of lead, and are reported at sample sizes sufficient to reliably estimate site-specific variability in lead levels.

We evaluate the importance of home range size and other movement-related traits as predictors of variability in contaminant levels among species with differing patterns of space use. In doing so, we aim to identify uncertainties arising from the use of spatially invariant approaches in the monitoring and assessment of chemically exposed wildlife. Our findings will inform the selection and sampling of species for understanding chemical threats to ecosystem health.

## Methods

### Species and site selection criteria

We limited our review to free-living, fully fledged birds for which the primary source of lead exposure was linked to terrestrial sources. As such, we excluded species with an aquatic trophic niche or primary lifestyle as defined by the AVONET trait database (Tobias et al. 2022). Lead exposure in aquatic foragers is often stochastic, driven by ingestion of contaminated shot or sediment (Pain et al. 2019). This makes site-level variability difficult to characterise reliably at typical sample sizes. Additionally, most waterbird and seabird studies do not target sites with independent environmental evidence of lead pollution. Moult timing and associated flightlessness also complicate the assumption that home range size reflects the spatial extent of lead exposure in feathers, though exogenous contamination post-growth is less affected by these constraints. Species associated with aquatic habitats but classified as terrestrial foragers under AVONET were considered for inclusion where they otherwise met our criteria. For example, *Branta canadensis*, classified within AVONET as a terrestrial herbivore and forager, were retained in the dataset.

As we were interested in how the use of space influences variability in lead exposure, we only included studies reporting site and species-specific data on feather lead concentrations. A site was considered a geographically discrete spatial unit that accounts for local environmental variation and enables replicable data collection, though we acknowledge that operational definitions vary across studies depending on research contexts, questions, and species. Given this uncertainty, we consider a geographically discrete site to be one in which the home range size of the sampled species could reasonably encompass the capture locations within that site. We therefore included sites of varying size as long as sampling took place within the same environmental context (e.g., within the same neighbourhood (McClelland et al. 2019)), omitting samples pooled across regions or lead pollution gradients (e.g., spread over large distances from emission sources (Cooper et al. 2017)).

Within studies, we only considered sites potentially contaminated with lead (e.g., urban areas, industrial areas, mining and smelting operations). We therefore excluded reference or background sites (e.g., rural and agricultural areas, national parks, wilderness areas (Deng et al. 2007) where spatial heterogeneity in environmental lead contamination was expected to be low. Finally, we excluded sites that did not meet a minimum sample size requirement of ten individuals. We did this to limit bias associated with the use of the coefficient of variation at small sample sizes.

### Feather lead review

We first searched Scopus and Web of Science (05/03/2026) for studies reporting feather lead concentrations in birds, using the query: *(“bird” OR “avian” OR “species” OR “wildlife”) AND (“lead” OR “Pb” OR “metal” OR “element”) AND (“feather” OR “plumage”)*, excluding instances where “lead” appeared only in the abstract and applying additional exclusionary terms for experimental studies (Supplementary Text S1). After removing duplicate records within and across databases, this yielded 2,060 unique studies, which were manually screened for relevance based on title and abstract and then assessed against our inclusion criteria above (Supplementary Fig. S2). Studies meeting these criteria were used for forward and backward citation searching, yielding a further 1,864 references filtered using the same procedure.

### Home range review

We then reviewed scientific literature on the home range of species for which site-specific feather lead data meeting the inclusionary criteria were available. For our review, home range was defined as the area routinely traversed by an individual in the course of its normal daily activities, including foraging, reproduction, and raising young (Powell and Mitchell 2012). We did not apply a specific mathematical criterion, and considered any numerical estimate intended to characterise the area of routine space use compatible with this definition.

In Scopus and Web of Science we used the following query to conduct a literature search of titles, abstracts and keywords: *“common name” OR “scientific name” AND “home range”*. The quantification of home range size was often supplementary to the research aims of the reviewed studies, and so we repeated this search on whole-article content using the same query in Google Scholar. A follow-up review was conducted using information on movement behaviours from Billerman et al. (2022).

Where feather lead data was available for a certain species, but home range data was not, we used estimates from closely related species as a proxy. Genus-level estimates were used for *Alcippe morrisonia* (from *A. davidi*), *Passer italiae* (from *P. domesticus* and *P. montanus*), *Pycnonotus jocosus* (from *P. goiavier*, *P. plumosus*, and *P. simplex*), while a family-level estimate was used for *Cyanoderma ruficeps* (from *Pomatorhinus ruficollis*).

### Data extraction

From studies reporting feather lead concentrations in birds with documented home ranges, we manually extracted site-specific data on the mean, variance (usually standard deviation or standard error) and sample size. Site characteristics, including primary pollution sources, were categorised into three broad groupings (urban-industrial, mining-smelting, military-hunting). We recorded coordinate information for each site based on location descriptions and site maps. Using land use data (2014–2016; based on the average publication date) from Copernicus Land Monitoring Service (Buchhorn et al. 2020), we calculated the percentage of built up cover within a 1 km radius of each approximate site location. We also recorded information on the methods used by each study, including the type of feather sampled and if the feathers were washed prior to analysis.

We manually extracted data from the literature on the home range of each species and the methods used in its measurement (e.g., observation, telemetry) and calculation (e.g., linear distances, kernel density estimates, minimum convex polygons). Where more than one method of calculation was reported, we used values derived from or most closely approximating to 95% kernel density estimates (Laver and Kelly 2008). Home range estimates were sourced from different studies and locations to populations from which the feather data was obtained.

To enable assessment of potential sources of variation in home range size, we recorded the season in which each home range estimate was obtained (breeding, non-breeding, multiple). We did not enforce inclusion criteria related to the timing of home range measurements, with two exceptions. First, where studies reported home range sizes for both breeding and non-breeding seasons, we used the breeding season estimate to better reflect movement behaviour during the moult period. Second, we excluded multi-season home range estimates for facultative migrants, specifically *Coragyps atratus* and *Cathartes aura*. We also estimated the percentage of built-up cover within a 1 km radius of each home range study site, using the same land use data and method described above.

We calculated the coefficient of variation using site-specific values for the mean and standard deviation of feather lead concentrations. The weighted mean of the home range estimates reported for each species was calculated using sample size as a weighting factor. Inverse variance weighting was not applicable due to the number of studies that did not report a measure of variance alongside home range values. To avoid biasing home range estimates towards the larger and less precise sample sizes of mark-recapture and observational methods, we set the weighting factor of estimates based on these methods to one.

Ecological trait data was extracted from AVONET (Tobias et al. 2022). We included information on body mass, migration, trophic level, and primary lifestyle. Partially migratory birds were reassigned to resident based on the description of the sampled populations. Here, the primary lifestyle of a bird is defined as the main locomotory niche used while foraging (Tobias et al. 2022). Only one species (*Hirundo rustica*) had a primary lifestyle categorised as aerial, and so it was merged with arboreal species (termed as insessorial in the AvoNet database) to create a single class of non-ground foraging birds for inclusion in the model selection process.

### Model selection

We determined the random effects structure prior to fixed effects selection. Intercept-only models established a site (χ² = 34.81, *p* < 0.001) and study (χ² = 56.49, *p* < 0.001) baseline via likelihood ratio tests. A species random intercept significantly improved fit over this baseline (χ² = 10.38, *p* = 0.001) and was retained in all subsequent candidate models. A phylogenetic covariance term was then added to the species model to test whether taxonomic relatedness explained variance beyond species identity. This was non-significant (χ² = 0.85, *p* = 0.36), and so the phylogenetic term was excluded on grounds of model parsimony.

We then constructed a series of linear mixed models using the site-specific coefficient of variation for feather lead concentrations as the response variable. Candidate predictors included home range size (ha), body mass (g), migration status (resident, migratory), trophic level (herbivore, omnivore, carnivore), primary lifestyle (terrestrial, generalist, arboreal-aerial), built area (%), context (military-hunting, mining-smelting, urban-industrial), feather type (contour, flight, tail, mixed), and feather treatment (washed, unwashed). Two-way interactions were included for all predictors. Continuous response and explanatory variables were log transformed where necessary to conform to model assumptions. Pairings of home range and body mass, primary lifestyle and trophic level, and feather treatment and feather type were not included in the same model as they were moderately to strongly associated based on Spearman’s (*r* > 0.5) and Cramer’s (v > 0.5) correlation coefficients. Interactions that were rank-deficient or exhibited near-zero variance across the dataset were also excluded.

Models were ranked using the Akaike information criterion corrected for small sample sizes (AICc). To avoid over-parameterisation, candidate models were limited to a maximum of five terms, approximating a degrees of freedom budget of 15 observations per term (Jenkins and Quintana-Ascencio 2020). The mean degrees of freedom cost per term in our global model was 1.74. The best-supported model was refitted using restricted maximum likelihood (REML) for final parameter estimation. Degrees of freedom for fixed effects were estimated using the Kenward–Roger approximation. Post hoc comparisons between groups were conducted using estimated marginal means with Tukey-adjusted pairwise contrasts, and Holm-adjusted tests for slope comparisons against zero.

Alongside the AICc model selection procedure, we conducted a grouped lasso as an independent assessment of variable importance across the same candidate space. The penalty lambda was selected by 10-fold cross-validation, using the most regularised value within one standard error of the minimum cross-validated error. At this lambda, the model was refit on 1,000 bootstrap resamples of the full dataset, and a retention score was calculated for each term group as the percentage of resamples in which it received a non-zero coefficient. The lasso was run independently of AICc selection and is interpreted separately.

### Model diagnostics

Model fit was assessed via standard residual diagnostics. To assess whether any individual observations exerted disproportionate influence on model parameter estimates, we calculated Cook’s distance for each observation, flagging the top 5% of values. We then repeated the full model selection procedure with these observations removed to determine whether retained variables were sensitive to their inclusion.

We next addressed the possibility that any relationship detected between home range size and variability in feather lead concentrations could reflect unresolved variance associated with site or study-level sampling design. First, we subset the data to sites and studies for which multiple species were sampled and fitted models treating site or study as a fixed effect, estimating the home range slope from within-group contrasts and comparing it against the full data estimate. Second, to assess whether home range was proxying for site or study-level differences in spatial scale, we compared site, study, and species variance components between an intercept-only mixed model and a model containing home range. Third, where detail was available in the source studies, we estimated site area directly and tested its correlation with species home range size using Spearman’s rank correlation. Using the same data, we compared site area against the total potential area covered by the home range sizes of species captured from that site.

We also evaluated whether variation in the conditions and contexts under which home range estimates were measured biased species-level values, using mixed models with species as a random intercept. We first calculated the intraclass correlation coefficient from an intercept-only model, restricted to species with three or more estimates, to partition variance within and between species. Second, we tested whether measurement season predicted home range size, restricted to species with estimates from both breeding and non-breeding seasons. Third, we tested whether built-up cover predicted home range size, restricted to species with three or more estimates, using the full dataset and then repeated after restricting data to developed landscapes exceeding 20% built cover (Zhou et al. 2015). Where seasonal or urbanisation effects were significant, we repeated our primary model selection procedure on the relevant subset (breeding season or above 20% built cover) to evaluate whether these effects influenced our main results.

Analyses were run in R 4.4.2. Random effects structures were evaluated using likelihood ratio tests in *lme4* (Bates et al. 2015), with degrees of freedom estimated using *lmerTest* (Kuznetsova et al. 2017). Model selection was conducted using *MuMIn* (Bartoń 2022), with *emmeans* for post hoc contrasts (Lenth 2025). The grouped lasso analysis used *grpreg* (Breheny and Huang 2015). Phylogenetic model building used *phyr* (Li et al. 2020). Phylogenetic data were sourced from *VertLife* (Jetz et al. 2012) and processed and visualised using *ape* (Paradis et al. 2004) and *ggtree* (Yu et al. 2017).

## Results

We found 44 studies that met the inclusion criteria and reported feather lead data from birds with documented home ranges (Supplementary Text S3). The average year of publication was 2014. These studies included data from 28 species across 101 unique sites, representing 3,065 individual lead measurements. Multiple averages were often reported from the same site, and so a total of 143 variation coefficients were calculated from this dataset.

**Fig. 1:**
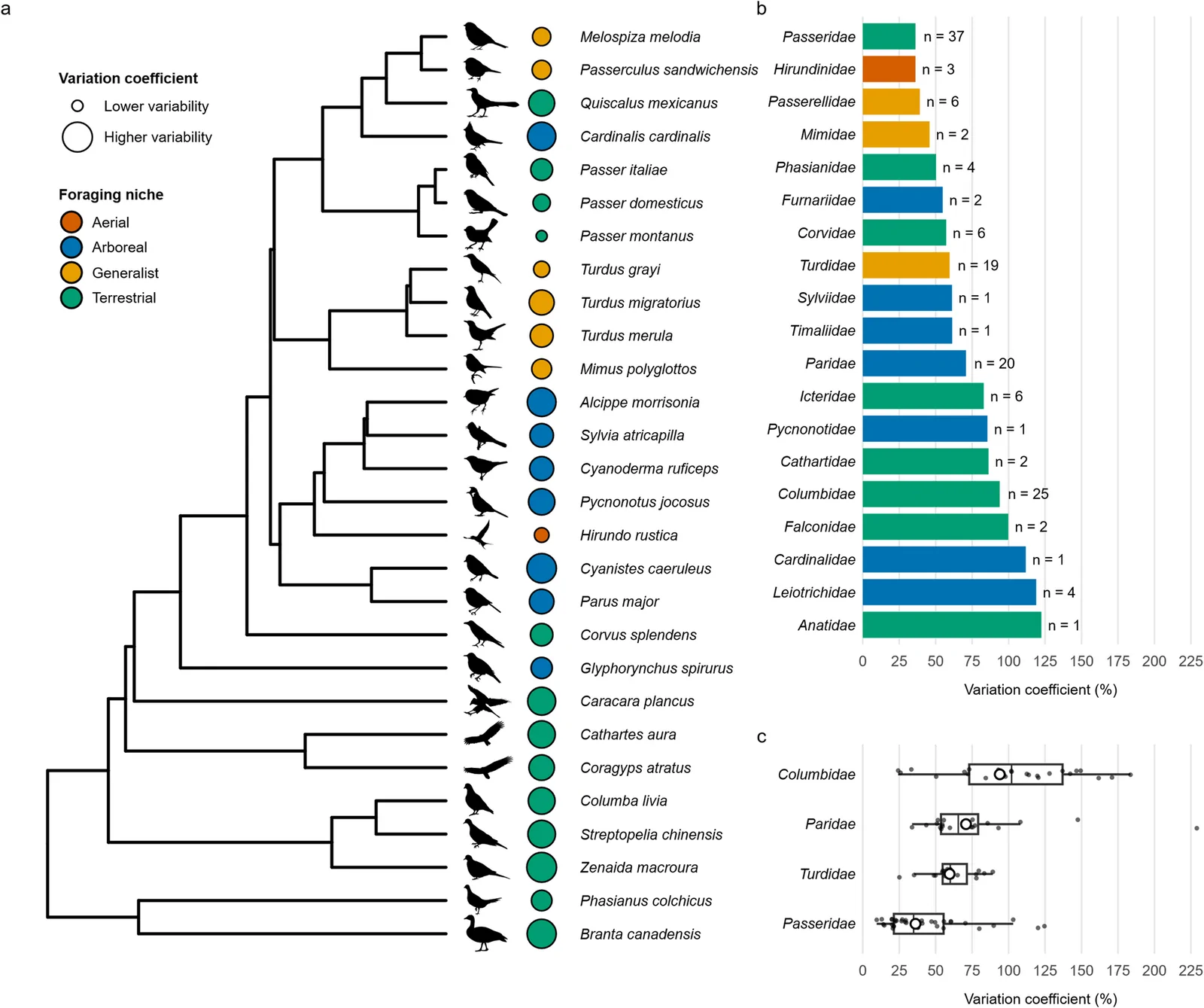
Geometric mean variation coefficients summarised by species (a) and family (b, c). Node distances reflect species relatedness, point size indicates relative variation coefficients, and colours designate primary lifestyle. Species silhouettes are sourced from PhyloPic.org

The most represented species were widely distributed birds commonly associated with urban environments (Table 1; Fig. 1), including Old World sparrows (Passeridae), pigeons and doves (Columbidae), tits (Paridae), and thrushes (Turdidae). Nearly half of the species represented were classified as occupying terrestrial foraging niche (46%), with smaller proportions of arboreal–aerial (33%) and generalist species (21%).

Most sites were located in urban–industrial (61%) and mining–smelting (33%) contexts, with a smaller proportion in military–hunting areas (6%). On average, built-up areas accounted for approximately 58% of land within a 1 km radius of sites. Most sites were located in the Northern Hemisphere (94%).

Relative variability in feather lead concentrations (Table 1; Fig. 1) was lowest in Passeridae (35%), Hirundinidae (35%), and Passerellidae (38%), and highest in Anatidae (122%), Leiotrichidae (118%), and Cardinalidae (111%). In the two most widely represented orders, variability was nearly twice as high in Columbiformes (93%) as in Passeriformes (51%).

**Table 1:** Variation coefficients (geometric mean and count), home range sizes (sample size weighted mean), feather lead concentrations (geometric mean), and average body mass (Tobias et al. 2022) of species included in this study

Common name	Scientific name	Variation coefficient (%)	Home range (ha)	Feather lead (mg/kg)	Body mass (g)
American Robin	*Turdus migratorius*	70.2 (*n* = 3)	859.1	12.72	78.5
Barn Swallow	*Hirundo rustica*	35.2 (*n* = 3)	120.1	55.7	17.9
Black Vulture	*Coragyps atratus*	81.7 (*n* = 1)	17872.8	3.45	1881.7
Canada Goose	*Branta canadensis*	121.5 (*n* = 1)	1935.5	1.65	2811.7
Clay-colored Thrush	*Turdus grayi*	35.2 (*n* = 1)	2.6	3.1	79.5
Crested Caracara	*Caracara plancus*	98.7 (*n* = 2)	1252.0	5.35	1078.6
Eurasian Blackbird	*Turdus merula*	58.4 (*n* = 15)	10.0	8.02	102.7
Eurasian Blackcap	*Sylvia atricapilla*	60.2 (*n* = 1)	5.0	3.57	16.7
Eurasian Blue Tit	*Cyanistes caeruleus*	123.1 (*n* = 3)	3.1	11.34	11.1
Eurasian Tree Sparrow	*Passer montanus*	28.1 (*n* = 11)	6.9	18.4	21.4
Great Tit	*Parus major*	63.1 (*n* = 17)	7.3	28.4	16.3
Great-tailed Grackle	*Quiscalus mexicanus*	81.9 (*n* = 6)	1508.2	2.5	160.5
House Crow	*Corvus splendens*	56.2 (*n* = 6)	49.7	19.06	292.6
House Sparrow	*Passer domesticus*	36.7 (*n* = 23)	21.2	5.34	26.5
Italian Sparrow	*Passer italiae*	54.1 (*n* = 3)	20.4	1.82	28.3
Morrison’s Fulvetta	*Alcippe morrisonia*	118.0 (*n* = 4)	3.2	42.35	15.3
Mourning Dove	*Zenaida macroura*	130.3 (*n* = 4)	1457.0	1.73	118.9
Northern Cardinal	*Cardinalis cardinalis*	110.8 (*n* = 1)	2.0	19.7	42.6
Northern Mockingbird	*Mimus polyglottos*	44.8 (*n* = 2)	3.8	13.69	48.5
Red-whiskered Bulbul	*Pycnonotus jocosus*	84.5 (*n* = 1)	14.6	3.48	29.5
Ring-necked Pheasant	*Phasianus colchicus*	49.2 (*n* = 4)	41.4	3.22	1120.3
Rock Pigeon	*Columba livia*	86.4 (*n* = 20)	135.5	6.02	354.2
Rufous-capped Babbler	*Cyanoderma ruficeps*	60.3 (*n* = 1)	2.7	26.11	10.3
Savannah Sparrow	*Passerculus sandwichensis*	41.1 (*n* = 1)	9.5	0.77	20.0
Song Sparrow	*Melospiza melodia*	37.6 (*n* = 5)	1.0	1.47	21.9
Spotted Dove	*Streptopelia chinensis*	97.8 (*n* = 1)	22.1	5.9	159.0
Turkey Vulture	*Cathartes aura*	88.9 (*n* = 1)	24152.5	2.69	1518.2
Wedge-billed Woodcreeper	*Glyphorynchus spirurus*	53.8 (*n* = 2)	4.1	0.97	14.6

**Fig. 2:**
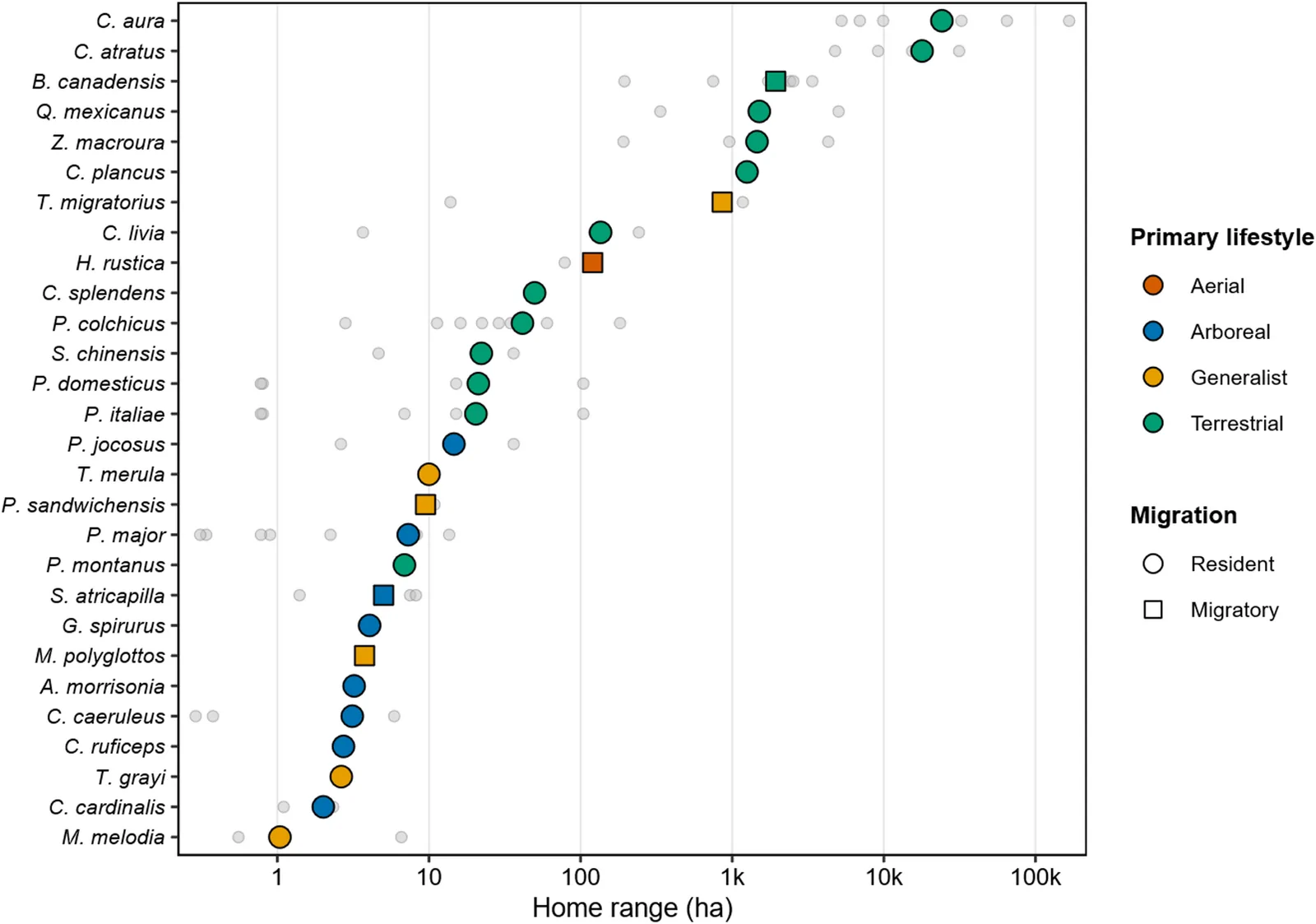
Weighted home range estimates for the reviewed bird species (in ascending order). Larger points indicate species weighted home range sizes. Smaller points indicate within-study weighted home range sizes for those same species. Point colour and shape indicate primary lifestyle and migratory behaviour of the included species

Home range estimates (Table 1; Fig. 2) for the 28 included species were sourced from 63 studies (Supplementary Text S4). There was substantial variation in home range size, including within the same study and population (Fig. 2). However, within-species variation was considerably smaller than between-species differences. Most species had weighted mean home range sizes below 10 ha (*n* = 12), with smaller numbers of species with sizes between 10 and 100 ha (*n* = 7), 100–1000 ha (*n* = 3), and above 1000 ha (*n* = 6).

With the site-specific coefficient of variation as the response variable and random effects of site, study, and species included, the best-supported model retained home range size, primary lifestyle, feather type, and an interaction between home range size and primary lifestyle (Table 2). These terms were present in all models within four AICc units of the best-supported model (Supplementary Table S5). Random effects variance was distributed across study (28.5%), site (28.3%), and species (6.8%), with species showing the largest reduction in variance relative to the intercept-only model (Supplementary Table S6). This is consistent with the fixed effects capturing predominantly species-level variation. Phylogenetic relatedness accounted for 10.3% of total random effects variance in the full phylogenetic model (Supplementary Table S7).

**Table 2:** Top ranked linear mixed model predicting site-specific variability in feather lead concentrations (coefficient of variation), with study, site and species as random effects

Main Effects					
Predictor	Coefficient	Std error	t-value	*p*-value	
Intercept	2.857	0.254	11.231	< 0.001	***
Ln(Home Range)	0.222	0.044	5.043	< 0.001	***
**Primary Lifestyle**					
Terrestrial (ref.)	-	-	-	-	
Generalist	0.481	0.339	1.42	0.169	
Arboreal-Aerial	1.701	0.359	4.742	< 0.001	***
**Feather Type**					
Contour (ref.)	-	-	-	-	
Wing	0.233	0.134	1.742	0.086	.
Tail	0.225	0.136	1.651	0.103	
Mixed	0.626	0.185	3.391	0.002	**
**Interactions**					
Ln(Home Range) × Terrestrial (ref.)	-	-	-	-	
Ln(Home Range) × Generalist	-0.077	0.088	-0.873	0.39	
Ln(Home Range) × Arboreal-Aerial	-0.459	0.116	-3.961	< 0.001	***

Random Effects			
Component	Variance	SD	Variance (%)
Study	0.065	0.256	28.5
Site	0.065	0.255	28.3
Species	0.016	0.125	6.8
Residual	0.084	0.289	36.4

Coefficients are based on log transformed variation coefficients and home range sizes, with Kenward-Roger degrees of freedom. Reference categories are terrestrial (primary lifestyle) and contour (feather type)

**Fig. 3:**
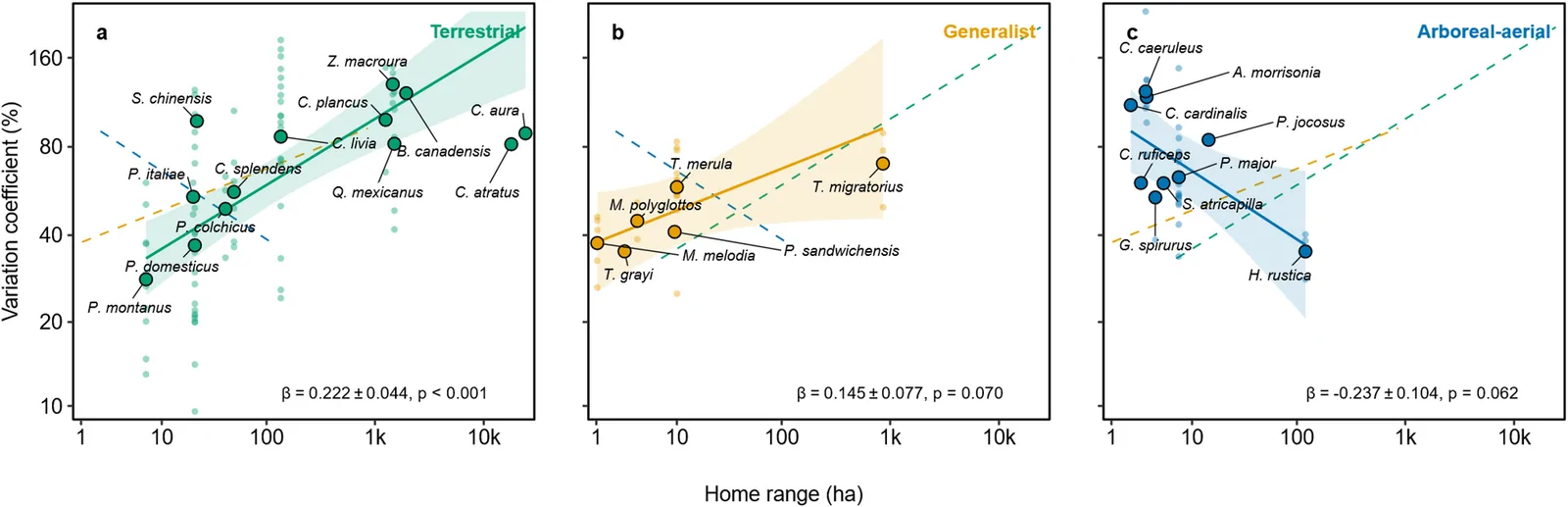
Top-ranked linear mixed model relating the coefficient of variation in feather lead (%) to home range size by primary lifestyle: terrestrial (a), generalist (b), arboreal-aerial (c). Solid lines show the fitted trend for each lifestyle; shaded bands show 95% confidence intervals; dotted lines show the fitted trends for the other two lifestyles. Large points show the mean log transformed variation coefficient for each species; smaller points show site-specific values. Coefficients were fitted on log transformed data and plotted on a linear scale. The arboreal-aerial category combines arboreal species with one aerial species

In the final model, a significant interaction between home range size and primary lifestyle (LRT: χ² = 15.604, df = 2, *p* < 0.001) indicated that the relationship between home range size and relative variability in feather lead concentrations differed between lifestyle groups (Table 2; Supplementary Table S8). Holm-adjusted slope tests (Supplementary Table S9) showed that relative variability increased significantly with home range size in terrestrial species (β = 0.222 ± 0.044, t = 5.043, *p* < 0.001), while slopes did not differ significantly from zero in generalist (β = 0.145 ± 0.077, t = 1.882, *p* = 0.070) or arboreal-aerial species (β = −0.237 ± 0.104, t = − 2.271, *p* = 0.062), with the latter trending negative (Fig. 3). Tukey-adjusted pairwise comparisons (Supplementary Table S9) confirmed significant differences in slopes between arboreal-aerial and terrestrial species (difference = 0.459 ± 0.116, t = 3.961, *p* = 0.001) and between arboreal-aerial and generalist species (difference = 0.382 ± 0.128, t = 2.985, *p* = 0.015), but not between terrestrial and generalist species (difference = 0.077 ± 0.088, t = 0.873, *p* = 0.661). However, home range values for arboreal-aerial and, to a lesser extent, generalist species were more positively skewed than those for terrestrial species (Fig. 3).

Variability in feather lead concentrations also differed by feather type (LRT: χ² = 12.797, df = 3, *p* = 0.005; Supplementary Table S9). Mixed-feather samples showed significantly higher variability than contour feathers (Tukey-adjusted β = 0.626 ± 0.185, t = 3.391, *p* = 0.008), whereas tail and wing feathers did not differ significantly from contour feathers (tail: β = 0.225 ± 0.136, t = 1.651, *p* = 0.358; wing: β = 0.233 ± 0.134, t = 1.742, *p* = 0.310). Tail, wing, and mixed feathers were similarly indistinguishable from each other (all pairwise *p* ≥ 0.150). Clearer differentiation of contour, wing and tail feathers was observed in the phylogenetic model (Supplementary Table S6), though direct comparison is limited by different model structures.

The grouped lasso (Supplementary Table S10) converged on the same two top-ranked terms as the AICc selection: the interaction between home range size and primary lifestyle (99.7% bootstrap retention) and feather type (99.6%). The main effects of home range size and primary lifestyle were also highly retained in the lasso (91.2% and 62.7%). Three additional terms showed high retention despite exclusion from the top AICc model, including a home range size and trophic level interaction (96.9%), a body mass and context interaction (79.1%), and a trophic level and built area interaction (74.9%).

Calculation of Cook’s distance identified eight observations in the top 5% of influential values for removal. Model selection rerun on the reduced dataset (*n* = 135) recovered an identical fixed effects structure to the primary analysis, indicating that influential observations did not drive variable retention (Supplementary Table S11).

The relationship between home range size and feather lead variability was robust to potential confounding by site extent and sampling design. Home range slopes estimated within multi-species sites and studies closely matched the full data marginal estimate, suggesting the relationship was not an artefact of between-site or between-study differences in spatial scale (within five multi-species sites: 0.088, 95% CI: −0.017, 0.193, *n* = 16; within eight multi-species studies: 0.060, 95% CI: −0.019, 0.140, *n* = 19; full data marginal: 0.080, 95% CI: 0.011, 0.149, *n* = 143). Adding home range to an intercept-only mixed model produced negligible changes in variance attributable to site (2.9% increase) and study (1.3% increase), while species variance decreased substantially (28.8%). This is consistent with home range operating as a species-level predictor rather than a proxy for site or study differences.

Species home range size and site area were not significantly correlated (Spearman: ρ = 0.243, *p* = 0.242, *n* = 25), providing limited evidence that larger sites were selected for far-ranging species. In 18 of the 25 sites for which area data could be extracted from the source studies, the potential home range coverage of the sampled species exceeded the reported site area (median ratio = 1.82). These reported site areas typically reflected the broader landscape context rather than the actual sampling footprint. For example, all seven sites where site area did exceed home range coverage involved short-ranging species (≤ 10 ha), six of which were sampled using mist nets, suggesting effective capture areas were considerably smaller than reported site boundaries.

Species-level home range estimates were robust to variation in measurement conditions across three diagnostic tests using mixed models with species as a random intercept. The intraclass correlation coefficient from an intercept-only model, restricted to species with three or more estimates (13 species, 139 observations), was 0.88, indicating that 88% of variance in home range size was attributable to between rather than within-species differences. Measurement season had no significant effect on home range size, with estimates from the non-breeding season not differing significantly from the breeding season (β = −0.282, df = 116.5, *p* = 0.222; restricted to 11 species with estimates from both seasons, 128 observations). Built-up cover was significantly and negatively related with home range size (β = −0.317, df = 126.4, *p* < 0.001), but this effect was not significant when restricted to landscapes exceeding 20% built cover (β = −0.252, df = 61.3, *p* = 0.517). Model selection repeated on the urban subset (*n* = 113) recovered a similar structure to the primary analysis, retaining feather type, primary lifestyle, and home range size, though the interaction between home range size and primary lifestyle was no longer retained (Supplementary Table S12).

## Discussion

In this study we reviewed and analysed associations between ecological traits and exposure variability in terrestrial birds. Our findings suggest that birds that are active over larger geographical areas tend to exhibit more variable levels of lead contamination than those with smaller home ranges. Furthermore, our results suggest associations between other ecological traits closely related to space use, including foraging niche and habitat preference (here encapsulated within the primary lifestyle variable), and divergent patterns of lead exposure within and between avian species. These findings highlight the importance of considering movement-related traits in the derivation and interpretation of wildlife exposure data.

### Home range

A growing body of research suggests that space use is a key determinant of animal exposure risk in the patchwork of contamination that characterises many modern ecosystems (Morrissey et al. 2024). Early conceptual models of this relationship indicated that while populations of far-ranging species often have lower overall levels of exposure, they are also more likely to contain individuals that exceed a specific exposure threshold (Marinussen and van der Zee 1996). This is corroborated by the patterns of lead exposure that we observed in birds with varying movement strategies. Species with the largest home range sizes more often had individuals with feather lead concentrations two or more times higher than their site average (Fig. 1). Average variation coefficients in *Columba livia* (86%) and *Zenaida macroura* (130%), for example, were two or more times higher than in *Passer montanus* (28%) and *Passer domesticus* (37%), which also had comparatively small home ranges (Fig. 2; Table 1). At least for ground foraging species (Fig. 3), our results here support the assumption that exposure variability scales with home range size in terrestrial birds. This has implications for species-specific exposure risks, as it implies that birds which are active over larger geographic areas are also more likely to encounter complex mixtures of chemicals with varying and potentially additive toxic effects (Anderson et al. 2023; Tartu et al. 2018). Alternatively, those with small home ranges are more susceptible to localised hotspots of contamination, especially if they overlap with patches of otherwise favourable habitat (Chow et al. 2005; Kompiš and Ballová 2021).

An increase in spatial variation in contaminant exposure with home range size has the potential to introduce a high degree of uncertainty in the monitoring and assessment of exposed wildlife. In our model selection process, the interaction between home range size and primary lifestyle ranked amongst the most important predictors of site-specific variability in feather lead concentrations (Supplementary Table S5). Home range size was also independently retained as a strong main effect by the grouped lasso (Supplementary Table S10). Larger home range sizes significantly increased exposure variability in birds occupying a terrestrial niche, with generalists showing a comparable but non-significant response (Fig. 3). This provides further evidence that actions to control for variability introduced by movement-related traits can improve the accuracy and reliability of exposure risk estimates (Chow et al. 2005), especially for species with specific foraging and habitat niches (e.g., ground foragers).

Integrating measures of home range size, core areas, and the temporal use of space within them, has been shown to additively improve extrapolations of internal contaminant burdens based on concentrations in environmental media (Hinton et al. 2019). Other studies have shown that sources of chemical pollution (e.g., pesticides) and patches of favourable habitat (e.g., croplands) often overlap, leading to more intense and frequent exposure events (Bro et al. 2015; Leeb et al. 2020; Lenhardt et al. 2015; Mayer et al. 2020). These studies suggest that spatially invariant approaches can lead to the systematic underestimation of exposure risk (Bæk et al. 2023; Bontrager et al. 2024; Hinton et al. 2019). Our results show that similar uncertainties apply to biomonitoring data gathered without consideration of the movement strategies of target species. This is particularly relevant for far-ranging species for which associations between core areas of activity and the underlying distribution of contamination are most unclear. Biomonitoring studies seeking to understand the impact of pollution on environmental, animal and human health over discrete geographic areas should increase sampling efforts to account for variability in chemical measurements introduced by larger home range sizes.

While not explicitly examined in our final model, interactions between closely related ecological traits such as home range, metabolic rate and body mass (Dial et al. 2008) can also influence spatial and temporal patterns of contaminant exposure and uptake in wildlife (Morrissey et al. 2024). Higher mass-specific metabolic rates are associated with a disproportionately higher intensity of resource utilisation relative to habitat patch size (Houston and McNamara 2014), which may in turn contribute to similar levels of exposure within small as opposed to large species. However, smaller species also detoxify and eliminate contaminants at a higher rate than larger species (Buekers et al. 2009; Kuo et al. 2022), leading to greater temporal fluctuations in their biological concentration following exposure events (Gillings et al. 2026). In birds, the time taken to replace and grow feathers is also related to body mass (Rohwer et al. 2009), introducing another potential source of variability in their chemical composition. Consequently, while the interaction between home range size and primary lifestyle outranked body mass in the model selection process, the importance of interrelated traits on exposure variability should not be discounted.

The migratory behaviour of the reviewed species was not included in the best ranked model, though was selected in similarly ranked models (delta < 4; Supplementary Table S5). Seasonal movements can introduce additional uncertainty in the identification of chemical sources in exposed wildlife. Studies of migratory birds often assume that chemicals contained in the endogenous component of feathers are closely related to exposure occuring at migratory grounds (Lavoie et al. 2014). This is because periods of moult and long-distance movement are energy intensive and rarely overlap (Kiat et al. 2019). Coupled with high levels of site fidelity to wintering and breeding grounds (Shimada et al. 2020), we might expect migration to have only a weak effect on site-specific variability in internal feather chemistry, as was observed here.

### Primary lifestyle

In the context of our analysis, primary lifestyle represents the predominant niche used by a species while foraging (Tobias et al. 2022). As primary lifestyle was closely associated with trophic level, its effect on variability in feather lead concentrations likely encompasses both the structural and functional aspects of this niche. Species lifestyle moderated the effect of home range on site-specific exposure variability, with arboreal-aerial species in particular exhibiting no significant increase in variation coefficients with home range size (Fig. 3). A clearer increase in exposure variability with respect to home range size in terrestrial and generalist foragers could be linked to the more direct exposure of these species to lead contaminated soil. High feather lead variability in arboreal species (Table 2), and its contrasting relationship with home range size relative to terrestrial and generalist species, might similarly relate to individual differences in movement strategies within vertically stratified habitats (Shepard et al., 2013). However, the majority of variation coefficients were derived from species with a terrestrial lifestyle, and data from arboreal-aerial and generalist species were less evenly distributed across home range sizes, which may have biased the relationships observed here.

Our analysis shows that the effect of smaller-scale spatial behaviours also warrants consideration in the interpretation of variability in wildlife exposure data. Ecological risk assessments are generally designed to capture variation in exposure pathways across species with differing ecological niches. As such, they are arguably more likely to target species with contrasting habitat preferences and foraging strategies than those with widely varying home range sizes (Rubach et al. 2011). Accordingly, traits including habitat preference, foraging strategy and trophic level are recognised for their influence in driving differential levels of exposure risk across species (Durkalec et al. 2022). There is also evidence that the relevance of these traits to differences in chemical sensitivities between species exceeds that of genetic factors such as phylogenetic relatedness (Bianchini and Morrissey 2020). Following this, we found only a weak and non-significant phylogenetic signal in our model of exposure variability (Supplementary Table S7). Spatial patterns of exposure related to the traits examined here do not appear to be phylogenetically constrained, at least within the reviewed bird species. This supports their application in ecological risk assessments targeting taxonomically diverse species assemblages (Van den Brink et al. 2011).

### Feather type

We divided feather types based on descriptions from the reviewed literature: contour (covering the body), wing (remiges), tail (rectrices), and mixed feathers. Variation coefficients were lower for samples of contour feathers than wing, tail, or mixtures of feather types (Table 2). This aligns with the consensus that contour feathers provide the most reliable indication of endogenous contaminant levels because they are more frequently and consistently moulted by birds (Furness et al. 1986; Jaspers et al. 2011). However, this difference was not significant compared to either wing or tail feathers, which also exhibited similar levels of variability in lead concentrations. Higher variability in mixed feathers may relate to inconsistencies in the type, number and size of feathers sampled between individuals from the same site, as well as the rate at which they accumulate contaminants such as lead (Low et al. 2020). Where possible, researchers should prioritise the sampling of contour feathers to limit unresolved variability related to feather type, and avoid using mixed feathers unless needed to minimise harm.

### Implications for wildlife exposure assessment

While our study only considered lead exposure in birds, the findings show how movement-related traits can strengthen ecological risk assessments targeting diverse wildlife and chemical contexts. In the many cases where the tracking of animal movements is unfeasible, traits such as home range size can be used to identify spatial overlaps between contaminant sources and the ecological drivers of animal movements, such as habitat structure and connectivity. Home range buffers are a highly simplified approximation of space use yet remain a practical method for linking populations to contaminant distributions and landscape-scale predictors of exposure risk (Fritsch et al. 2025; Fuentes et al. 2024; Taylor et al. 2023). External contaminant concentrations averaged within these spatial buffers show the strongest affinity to measurements from biota when their size is adjusted to specific species and landscape characteristics (Fritsch et al. 2012). Even coarse measurements of home range size are ecologically relevant to exposure levels in wildlife.

In highly mobile species where movement patterns are more uncertain, home range estimates can still be used in combination with geospatial datasets and temporal information to understand how landscape-scale factors influence exposure risk. For example, Fuentes et al. (2024) found that pesticide detection rates in Montagu’s harrier (*Circus pygargus*) nestlings were negatively associated with the proportion of organic farms within the home range of this species. Similarly, Fernández-Vizcaíno et al. (2023) detected residues of the fungicide tebuconazole in red-legged partridges (*Alectoris rufa*), but only for flocks that had home ranges intersecting recently sown cereal fields. The increasing spatial and temporal resolution of remotely sensed data will continue to expand the applicability of movement data and related traits to understanding chemical risks to far-ranging species.

Researchers can also apply the traits examined here to guide sampling protocols and analytical methods in wildlife biomonitoring studies. Far-ranging and migratory species, or those with complex foraging behaviours, may require more comprehensive screening approaches to account for greater uncertainty in chemical risks. For example, despite targeting non-agricultural areas, Anderson et al. (2023) reported elevated concentrations of neonicotinoid insecticides in American robins (*Turdus migratorius*) and red-winged blackbirds (*Agelaius phoeniceus*). These species have large home ranges which bring them into more frequent contact with diverse land uses, including agricultural areas contaminated with neonicotinoids (Anderson et al. 2023). Examples such as this show that while highly mobile and migratory species are at greatest risk of interaction with multiple chemical stressors, the full extent of these interactions are likely underestimated due to cost and time restrictions on the number of analytes that can be reasonably targeted by most studies.

Finally, our findings demonstrate the utility of sedentary species with restricted home ranges as biomonitors of environmental contamination and sentinels of chemical threats to ecosystem health. Among ground and generalist foragers, bird species with small home ranges consistently showed the lowest levels of site-specific exposure variability (Table 1). The restricted spatial activity of these birds facilitates cross-species extrapolation of exposure risks provided that other taxa are exposed over comparable scales of contamination. For example, Gillings et al. (2024a) documented strong correlations between blood lead levels in house sparrows (*Passer domesticus*) and young children residing in the same neighbourhoods. These links were spatially robust despite marked differences in habitat use and movement patterns among children and house sparrows. This demonstrates that appropriately selected species can serve as reliable indicators of ecosystem-wide chemical risks when their spatial ecology is considered in relation to underlying contaminant distributions.

### Limitations

Spatial variability in exposure arises from both the irregular use of space by a species and the uneven distribution of contamination within that space. In this study, we explored the effect of movement-related traits on variability in feather lead concentrations at a site-specific scale. While we considered factors such as the density of built up areas and varying anthropogenic lead sources, we did not control for differences in spatial patterns of lead contamination between sites. Most studies did not report site-specific soil lead data, and so it is unclear to what extent environmental heterogeneity, as opposed to behavioural traits, influenced our results. In our model, the random effects of study and site together accounted for a substantial proportion of residual variance after accounting for fixed effects (Table 2). Some of this variance is likely attributable to the different environmental contexts and sources of contamination examined by the reviewed studies.

In our analysis, we assumed home range size to be constant for a given species. This is clearly not the case, as estimates of species-specific home range sizes varied widely, even within the same population. There is a lack of consensus on the methods used to derive home range size which can lead to large variations in estimates from the same data (Laver and Kelly 2008). What constituted a site also differed between studies, introducing uncertainty about whether sites meaningfully reflected the spatial scale of exposure. However, diagnostic checks indicated that the observed relationship between home range size and feather lead variability was unlikely to reflect site or study-level confounding, and more likely captures a genuine species-level effect.

Furthermore, home range size and animal movements within this range are moderated by a number of biotic and abiotic factors, including habitat patch size, resource availability, predator-prey interactions and climate (Rolando 2002). Urban wildlife populations, for example, often have smaller home ranges than their rural counterparts, likely due to shifts in food sources and availability, and habitat structure (McGowan 2001; Šálek et al. 2015). A number of these factors, including sex, could not easily be controlled for due to inconsistent reporting in the reviewed studies. As such, our home range estimates do not account for the multiple ecological factors which may influence the movement strategies of the lead exposed populations reviewed here. Where we were able to control for variability in home range estimates, within-species values were relatively stable (ICC = 0.88) and showed limited seasonal variation, with the primary model structure remaining robust to restriction of the data to built-up landscapes.

The focus on feathers as opposed to internal biological tissues is debatable due to the number of biotic and abiotic factors that can influence variability in feather lead concentrations (Jaspers et al. 2019; Low et al. 2020). However, destructively sampled tissues such as kidneys and livers are rarely taken in numbers sufficient to reliably estimate site-specific levels of exposure variability. This problem is not as common in studies using blood samples, yet these more often have concentrations below analytical limits of detection which can strongly bias variance.

The use of the coefficient of variation as a measure of site-specific variability in feather lead concentrations also has its disadvantages. Low sample sizes and near zero values can skew variation coefficients and overestimate variability in sample pools. To control for this, we set a minimum site sample size of ten individuals and limited our analysis to contaminated sites with a greater potential for elevated feather lead concentrations. More robust measures of relative variability are available yet are rarely calculable from the most commonly reported summary statistics.

Our strict inclusion criteria may have limited sample sizes across some categorical variables, contributing, for example, to the underrepresentation of the military-hunting context and the partial confound between feather washing and feather type. Similarly, a narrower spread of home range sizes for arboreal-aerial and generalist species likely reduced power to detect reliable slopes within these groups, with both slope estimates bordering on marginal significance. The generalist slope did not differ significantly from that of terrestrial species, suggesting a similar positive relationship masked by insufficient power. The negative arboreal-aerial trend should be interpreted with caution given the disproportionate influence of the single aerial species, which had a comparatively large home range size and low feather lead variability. That home range size was robustly retained as a main effect by the grouped lasso independent of this interaction nonetheless reinforces its importance as a predictor of exposure variability across species.

## Conclusion

Globally, the scale and complexity of pollution is expanding (Naidu et al. 2021) and chemical threats to human, animal, and environmental health are becoming increasingly linked (Gillings et al. 2024a; Rabinowitz and Conti 2013). Within this emerging global paradigm, the irregular and uneven use of space by wildlife further challenges our ability to monitor and manage chemical threats across taxa and ecological contexts. To better understand how animal movements contribute to spatially variable patterns of exposure, we conducted a meta-analysis of environmental and ecological correlates of site-specific variability in avian feather lead concentrations, with a focus on movement-related behavioural traits. Our research supports the assumption that variation in exposure among some terrestrial birds is associated with a species' use of space within an area defined by its home range. Progress towards integrating animal movement behaviours into ecological risk assessments is hampered by a lack of data and uncertainties surrounding their generalisability and toxicological relevance (Morrissey et al. 2024). Our findings highlight a set of movement-related traits that are well documented, generalisable across taxa, and, in the absence of spatially explicit tracking data, can help constrain exposure variability attributable to species-specific movement behaviours.

## Supplementary Information

Below is the link to the electronic supplementary material.

Supplementary Material 1

Supplementary Material 2

## Data Availability

Data used in this study are available in the supplementary materials.
