# Supplementary material for: Home range size and foraging niche predict lead exposure variability in terrestrial birds
Source: Ecotoxicology. 2026 Jul 2;35(6):136. doi: 10.1007/s10646-026-03123-7 (PMC13328157; doi:10.1007/s10646-026-03123-7)
Supplement: Supplementary file 1 — Supplementary Material 1 [file 10646_2026_3123_MOESM1_ESM.docx]

**Supplementary Information for:**

**Title:** Home range size and foraging niche predict lead exposure variability in terrestrial birds

**Authors**: Max M Gillings^1^*, Riccardo Ton^1,2^, Simon C Griffith^1^

**Affiliations:**

^1^School of Natural Sciences, Faculty of Science and Engineering, Macquarie University, Sydney, New South Wales 2109, Australia.

^2^Cavanilles Institute of Biodiversity and Evolutionary Biology, University of Valencia, Valencia 46980, Spain.

*Corresponding author email: [Max.Mclennan-Gillings@mq.edu.au](mailto:Max.Mclennan-Gillings@mq.edu.au).

**This Supplementary Data file includes:**

Supplementary Materials S1–S12

**Supplementary Text S1.** Full Scopus database query for the systematic review of articles reporting feather lead concentrations in terrestrial birds, adapted for use in Web of Science. Wildcards were applied to search terms where supported by each database. The query excluded experimental dosing studies.

*(TITLE-ABS-KEY ("bird" OR "avian" OR "species" OR "wildlife") AND TITLE ("lead") OR KEY ("lead") OR TITLE-ABS-KEY ("Pb" OR "metal" OR "element") AND TITLE-ABS-KEY ("feather" OR "plumage") AND NOT TITLE-ABS-KEY ("dosing" OR "dose" OR "supplement" OR "trial" OR "treatment" OR "administer" OR "exposure experiment"))*

**Supplementary Figure S2.** PRISMA flow diagram showing the literature search and screening process.


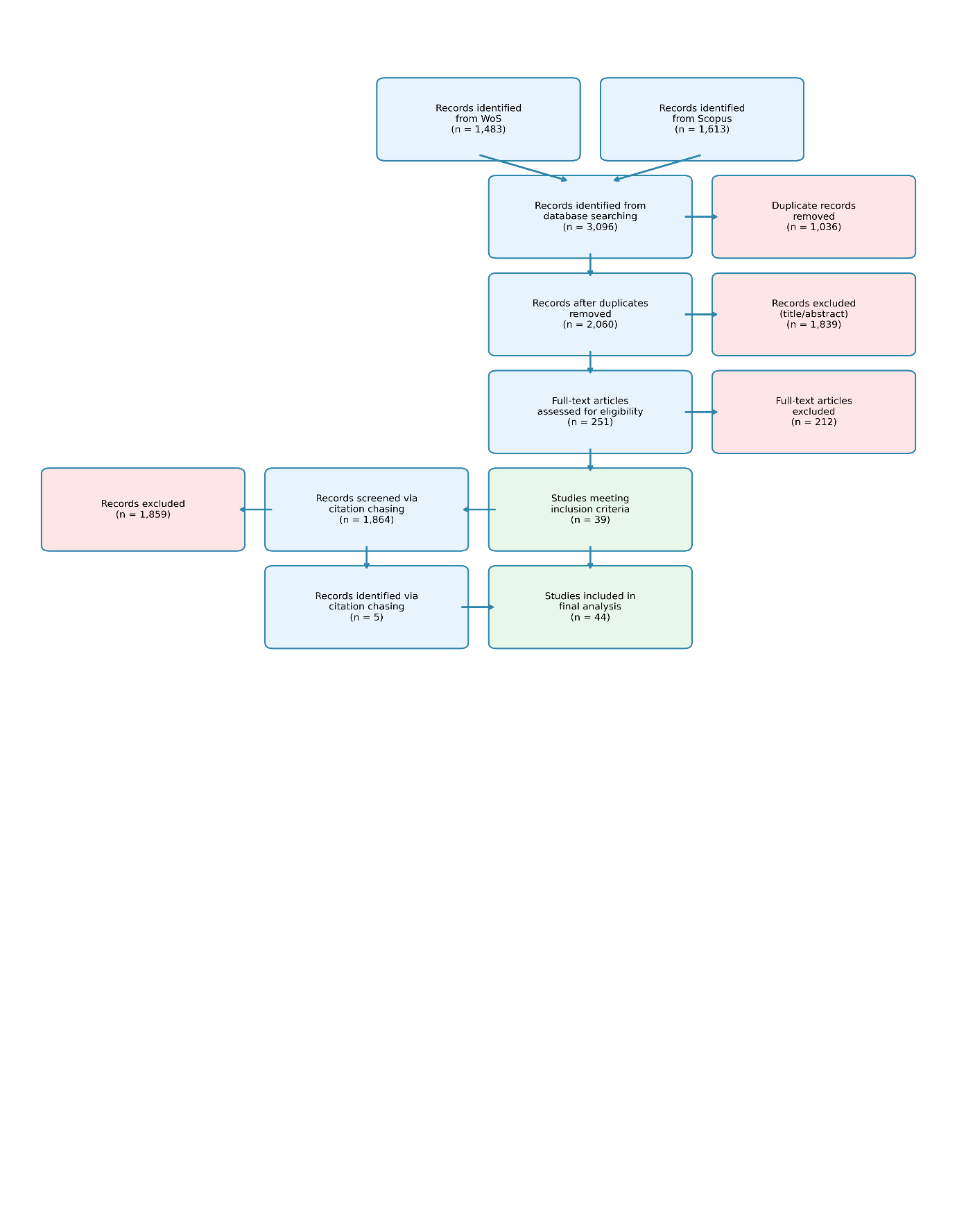


**Supplementary Text S3.** Reference list for the systematic literature review of feather lead concentrations in terrestrial birds.

1. Bargar T. Assessment of bird exposure to lead at Tyndall and Beale Air Force Bases, 2016–17. *US Geological Survey Open-File Report 2018-5164*. Reston, VA; 2019.

2. Bichet C, Scheifler R, Cœurdassier M, Julliard R, Sorci G, Loiseau C. Urbanization, trace metal pollution, and malaria prevalence in the house sparrow. *PLoS ONE* 2013, 8(1), e53866.

3. Burger J, Kennamer RA, Brisbin IL, Gochfeld M. Metal levels in mourning doves from South Carolina: potential hazards to doves and hunters. *Environmental Research* 1997, 75(2), 173–186.

4. Costa RA, Petronilho JMS, Soares AMVM, Vingada JV. The use of passerine feathers to evaluate heavy metal pollution in central Portugal. *Bulletin of Environmental Contamination and Toxicology* 2011, 86(3), 352–356.

5. Dai W, He C, Goodale E, Cao J, Jiang A, Su T, Pagani-Núñez E, Leng X. Behavioral assessment of heavy metal pollution effects on birds unveils species-specific responses to different elements. *Environmental Monitoring and Assessment* 2025, 197(12), 1375.

6. Dauwe T, Bervoets L, Jaspers V, Pinxten R, Blust R, Eens M. Great and blue tit feathers as biomonitors for heavy metal pollution. *Ecological Indicators* 2002, 1(4), 227–234.

7. Ding J, Wang S, Yang W, Zhang H, Yu F, Zhang Y. Tissue distribution and association of heavy metal accumulation in a free-living resident passerine bird tree sparrow *Passer montanus*. *Environmental Pollution* 2023, 316, 120547.

8. Ding J, Yang W, Wang S, Zhang H, Yang Y, Bao X, Zhang Y. Effects of environmental metal pollution on reproduction of a free-living resident songbird, the tree sparrow (*Passer montanus*). *Science of the Total Environment* 2020, 721, 137674.

9. Ding J, Yang W, Wang S, Zhang H, Zhang Y. Does environmental metal pollution affect bird morphometry? A case study on the tree sparrow *Passer montanus*. *Chemosphere* 2022, 295, 133947.

10. Durkalec M, Martínez-Haro M, Nawrocka A, Pareja-Carrera J, Smits JEG, Mateo R. Factors influencing lead, mercury and other trace element exposure in birds from metal mining areas. *Environmental Research* 2022, 212, 113575.

11. Eens M, Pinxten R, Verheyen RF, Blust R, Bervoets L. Great and blue tits as indicators of heavy metal contamination in terrestrial ecosystems. *Ecotoxicology and Environmental Safety* 1999, 44(1), 81–85.

12. El-Shabrawy NM, Kamel AM, Goda AS, Donia GR, Salah-Eldein AM. Comparison of heavy metals concentration in different tissues of four wild bird species. *Advances in Animal and Veterinary Sciences* 2022, 10(2), 307–315.

13. Frantz A, Pottier M-A, Karimi B, Corbel H, Aubry E, Haussy C, Gasparini J, Castrec-Rouelle M. Contrasting levels of heavy metals in the feathers of urban pigeons from close habitats suggest limited movements at a restricted scale. *Environmental Pollution* 2012, 168, 23–28.

14. Fritsch C, Coeurdassier M, Faivre B, Baurand P-E, Giraudoux P, van den Brink NW, Scheifler R. Influence of landscape composition and diversity on contaminant flux in terrestrial food webs: a case study of trace metal transfer to European blackbirds *Turdus merula*. *Science of the Total Environment* 2012, 432, 275–287.

15. Fritsch C, Jankowiak Ł, Wysocki D. Exposure to Pb impairs breeding success and is associated with longer lifespan in urban European blackbirds. *Scientific Reports* 2019, 9(1), 486.

16. Geens A, Dauwe T, Bervoets L, Blust R, Eens M. Haematological status of wintering great tits (*Parus major*) along a metal pollution gradient. *Science of the Total Environment* 2010, 408(5), 1174–1179.

17. Grue CE, O'Shea TJ, Hoffman DJ. Lead concentrations and reproduction in highway-nesting barn swallows. *The Condor* 1984, 86(4), 383–389.

18. Grunst ML, Grunst AS, Pinxten R, Bervoets L, Eens M. Carotenoid- but not melanin-based plumage coloration is negatively related to metal exposure and proximity to the road in an urban songbird. *Environmental Pollution* 2020, 256, 113473.

19. Guzmán-Velasco A, Ramírez-Cruz JI, Ruiz-Aymá G, Rodríguez-Sánchez IP, Garza-Ocañas L, Treviño-Alvarado VM, González-Rojas JI, Olalla-Kerstupp A. Great-tailed grackles (*Quiscalus mexicanus*) as biomonitors of atmospheric heavy metal pollution in urban areas of Monterrey, Mexico. *Bulletin of Environmental Contamination and Toxicology* 2021, 106(6), 983–988.

20. Haddadi Y, Chahlaoui A, Taouraout A. Feathers of the house sparrow (*Passer domesticus*) as a non-invasive tool for assessing urban and industrial metal pollution in Meknes, Morocco. *Bulletin of Environmental Contamination and Toxicology* 2025, 115(6), 73.

21. Hamidian AH, Saberi SMJA, Ashrafi S, Moghaddasi L. Using house sparrow in biomonitoring lead in Tehran air pollution. *Urban Ecosystems* 2023, 26(4), 895–903.

22. Hoff Brait CH, Antoniosi Filho NR. Use of feathers of feral pigeons (*Columba livia*) as a technique for metal quantification and environmental monitoring. *Environmental Monitoring and Assessment* 2011, 179(1), 457–467.

23. Hoynes K, Holland AE, Bryan AL, Kupferman CA, Beasley JC. Trace elements and heavy metals in black vultures (*Coragyps atratus*) and turkey vultures (*Cathartes aura*) in the southeastern United States. *Environmental Science and Pollution Research* 2024, 31(6), 9000–9010.

24. Innangi M, De Rosa D, Danise T, Fozzi I, Giannotti M, Izzo M, Trifuoggi M, Fioretto A. Analysis of 11 trace elements in flight feathers of Italian sparrows in southern Italy: a study of bioaccumulation through age classes, variability in three years of sampling, and relations with body condition. *Science of the Total Environment* 2019, 651, 2003–2012.

25. Iqbal F, Ayub Q, Wilson R, Song BK, Talei A, Yeong KY, Hermawan AA, Fahim M, Rahman S. Monitoring of heavy metal pollution in urban and rural environments across Pakistan using house crows (*Corvus splendens*) as bioindicator. *Environmental Monitoring and Assessment* 2021, 193(4), 237.

26. Janaydeh M, Ismail A, Zulkifli SZ, Bejo MH, Aziz NAA, Taneenah A. The use of feather as an indicator for heavy metal contamination in house crow (*Corvus splendens*) in the Klang area, Selangor, Malaysia. *Environmental Science and Pollution Research* 2016, 23(21), 22059–22071.

27. Janssens E, Dauwe T, Bervoets L, Eens M. Heavy metals and selenium in feathers of great tits (*Parus major*) along a pollution gradient. *Environmental Toxicology and Chemistry* 2001, 20(12), 2815–2820.

28. Lester MB, van Riper C. The distribution and extent of heavy metal accumulation in song sparrows along Arizona's upper Santa Cruz River. *Environmental Monitoring and Assessment* 2014, 186(8), 4779–4791.

29. Li M, Nabi G, Sun Y, Wang Y, Wang L, Jiang C, Cao P, Wu Y, Li D. The effect of air pollution on immunological, antioxidative and hematological parameters, and body condition of Eurasian tree sparrows. *Ecotoxicology and Environmental Safety* 2021, 208, 111755.

30. Liu S, Mtemi WM, Zhou T, Pan J, Jiang A. Multiple trace elements exposure of grey-cheeked fulvettas *Alcippe morrisonia*, a nuclear member in bird mixed-species flocks, and implications for bioindicator. *Ecotoxicology and Environmental Safety* 2022, 244, 114063.

31. Loera Y, Gruppi C, Swing K, Campbell-Staton SC, Milá B, Smith TB. Heavy metal contamination in birds from protected regions in the Amazon. *Environmental Toxicology and Chemistry* 2024, 43(12), 2601–2607.

32. McClelland SC, Durães Ribeiro R, Mielke HW, Finkelstein ME, Gonzales CR, Jones JA, Komdeur J, Derryberry E, Saltzberg EB, Karubian J. Sub-lethal exposure to lead is associated with heightened aggression in an urban songbird. *Science of the Total Environment* 2019, 654, 593–603.

33. Nam D-H, Lee D-P, Koo T-H. Monitoring for lead pollution using feathers of feral pigeons (*Columba livia*) from Korea. *Environmental Monitoring and Assessment* 2004, 95(1), 13–22.

34. Nava-Diaz R, Hoogesteijn AL, Erosa MD, Febles JL, Mendez-Gonzalez RM. Comparative study of lead concentration in feathers of urban and rural passerines in Merida, Mexico. *Bulletin of Environmental Contamination and Toxicology* 2015, 95(4), 470–474.

35. Piroutková M, Husáková L, Kováčik J, Patočka J. Great tit rather than blue tit indicates metallic pollution: a multi-element analysis around nickel smelter. *Atmospheric Pollution Research* 2026, 17(4), 102850.

36. Riccardi A, Douglass KR, Jackson VS, Dehnert GK, Herbst H, Grewe F, Walker M, Magle S, Murray MH, Adams H, Brook CE, Ruhs EC. Impacts of urbanization on the health of American robins (*Turdus migratorius*) in Chicagoland. *bioRxiv* 2024, 2024.11.24.625046.

37. Sawicka-Kapusta K, Kozłowski J, Sokołowska T. Heavy metals in tits from polluted forests in southern Poland. *Environmental Pollution Series A, Ecological and Biological* 1986, 42(4), 297–310.

38. Scheifler R, Cœurdassier M, Morilhat C, Bernard N, Faivre B, Flicoteaux P, Giraudoux P, Noël M, Piotte P, Rieffel D, de Vaufleury A, Badot PM. Lead concentrations in feathers and blood of common blackbirds (*Turdus merula*) and in earthworms inhabiting unpolluted and moderately polluted urban areas. *Science of the Total Environment* 2006, 371(1), 197–205.

39. Silva LTR, Filho EFO, Kunst TH, Rolim VPM, Silva JSA, Regueira RFS, Paim APS, Soares PC, Oliveira AAF. Heavy metal concentrations in free-living southern caracaras (*Caracara plancus*) in the northeast region of Brazil. *Acta Scientiae Veterinariae* 2017, 45(1), 8.

40. Solonen T, Lodenius M, Tulisalo E. Metal levels of feathers in birds of various food chains in southern Finland. *Ornis Fennica* 1999, 76(1), 25–32.

41. Tajchman K, Drabik K, Ukalska-Jaruga A, Janiszewski P, Spustek D, Wengerska K. The screening method for use of wild pheasant feathers in the monitoring of environmental pollution with heavy metals. *Scientific Reports* 2023, 13(1), 6540.

42. Tkachenko H, Kurhaluk N, Hetmański T, Włodarkiewicz A, Tomin V. Changes in energetic metabolism and lysosomal destruction in the skeletal muscle and cardiac tissues of pigeons (*Columba livia* f. urbana) from urban areas of the northern Pomeranian region (Poland). *Ecotoxicology* 2021, 30(6), 1170–1185.

43. Tsipoura N, Burger J, Newhouse M, Jeitner C, Gochfeld M, Mizrahi D. Lead, mercury, cadmium, chromium, and arsenic levels in eggs, feathers, and tissues of Canada geese of the New Jersey Meadowlands. *Environmental Research* 2011, 111(6), 775–784.

44. Yao T, Zhu G, Zhang Y, Yan P, Li C, de Boer WF. Bird's feather as an effective bioindicator for detection of trace elements in polymetallic contaminated areas in Anhui Province, China. *Science of the Total Environment* 2021, 771, 144816.

**Supplementary Text S4.** Reference list for literature review on home range sizes in terrestrial birds.

1. Arias Medellin LA. Disentangling the roles of pollination and seed dispersal on the demography of a tropical understory herb (*Heliconia tortuosa*) in a fragmented landscape. Thesis; 2022.
2. Assandri G, Morganti M, Bogliani G, Pulido F. The value of abandoned olive groves for blackcaps (*Sylvia atricapilla*) in a Mediterranean agroecosystem: a year-round telemetry study. *European Journal of Wildlife Research* 2017, 63(1), 26.
3. Ausprey IJ, Rodewald AD. Post-fledging dispersal timing and natal range size of two songbird species in an urbanizing landscape. *The Condor* 2013, 115(1), 102–114.
4. Avery ML, Humphrey JS, Daughtery TS, Fischer JW, Milleson MP, Tillman EA, Bruce WE, Walter WD. Vulture flight behavior and implications for aircraft safety. *Journal of Wildlife Management* 2011, 75(7), 1581–1587.
5. Basso E, Navarrete C, Riquelme-Ortiz A, Suárez C, Barber DR, Bildstein KL, Graña Grilli M, Lambertucci SA. Central Chile comprises a previously unknown nonbreeding area for the migratory population of turkey vulture (*Cathartes aura ruficollis*) breeding in the northwestern Argentine Patagonia. *Emu — Austral Ornithology* 2024, 124(2), 206–210.
6. Beat N-D, Martin UG. Post-fledging range use of great tit (*Parus major*) families in relation to chick body condition. *Ardea* 2008, 96(2), 181–190.
7. Benson TJ, Ward MP, Lampman RL, Raim A, Weatherhead PJ. Implications of spatial patterns of roosting and movements of American robins for West Nile virus transmission. *Vector-Borne and Zoonotic Diseases* 2012, 12(10), 877–885.
8. Biedenweg DW. Time and energy budgets of the mockingbird (*Mimus polyglottos*) during the breeding season. *The Auk* 1983, 100(1), 149–160.
9. Björklund M, Westman B. Mate-guarding in the great tit: tactics of a territorial forest-living species. *Ornis Scandinavica* 1986, 17(2), 99–105.
10. Caprio E, Rolando A. Management systems may affect the feeding ecology of great tits *Parus major* nesting in vineyards. *Agriculture, Ecosystems & Environment* 2017, 243, 67–73.
11. Clark L. Rock pigeon use of livestock facilities in northern Colorado: implications for improving farm bio-security. *Human-Wildlife Interactions* 2011.
12. Coleman JS. Home range, habitat use, behavior, and morphology of the Gettysburg vultures. Thesis, Virginia Polytechnic Institute and State University; 1985.
13. Coleman JS, Fraser JD. Habitat use and home ranges of black and turkey vultures. *Journal of Wildlife Management* 1989, 53(3), 782–792.
14. Daniel LG, Martha JD. Influence of foraging and roosting behavior on home-range size and movement patterns of savannah sparrows wintering in south Texas. *The Wilson Bulletin* 2005, 117(1), 63–71.
15. Darrah AJ, Smith KG. Ecological and behavioral correlates of individual flocking propensity of a tropical songbird. *Behavioral Ecology* 2014, 25(5), 1064–1072.
16. Eberhardt LE, Anthony RG, Rickard WH. Movement and habitat use by Great Basin Canada goose broods. *Journal of Wildlife Management* 1989, 53(3), 740–748.
17. Ferry C, Frochot B, Leruth Y. Territory and home range of the blackcap (*Sylvia atricapilla*) and some other passerines, assessed and compared by mapping and capture-recapture. *Studies in Avian Biology* 1981, 6(1), 29.
18. Fülöp A, Lukács D, Barta Z. Space use of wintering Eurasian tree sparrows (*Passer montanus*) in a semi-urban area: a radiotelemetry-based case study. *Ornis Hungarica* 2022, 30(2), 124–133.
19. Gatti RC, Dumke RT, Pils CM. Habitat use and movements of female ring-necked pheasants during fall and winter. *Journal of Wildlife Management* 1989, 53(2), 462–475.
20. Genovesi P, Besa M, Toso S. Habitat selection by breeding pheasants *Phasianus colchicus* in an agricultural area of northern Italy. *Wildlife Biology* 1999, 5(4), 193–201.
21. Gordon CE. Movement patterns of wintering grassland sparrows in Arizona. *The Auk* 2000, 117(3), 748–759.
22. Groepper S, Gabig P, Vrtiska M, Gilsdorf J, Hygnstrom S, Joseph R. Population and spatial dynamics of resident Canada geese in southeastern Nebraska. *Human-Wildlife Conflicts* 2008, 2.
23. H Jeffrey H, George ML, William JB. Winter habitat use and survival of female ring-necked pheasants (*Phasianus colchicus*) in southeastern North Dakota. *The American Midland Naturalist* 2000, 143(2), 463–480.
24. Halliburton R, Mewaldt LR. Survival and mobility in a population of Pacific coast song sparrows (*Melospiza melodia gouldii*). *The Condor* 1976, 78(4), 499–504.
25. Harris ME, Hobson KA, Morrissey CA. Barn swallows (*Hirundo rustica*) and tree swallows (*Tachycineta bicolor*) select wetlands in agriculturally intensive landscapes, as revealed by GPS tracking. *Ornithological Applications* 2024, 126(3), duae012.
26. Havlíček J, Riegert J, Fuchs R. A comparison of foraging-range sizes, flight distances and foraging habitat preferences in urban and rural house sparrow (*Passer domesticus*) populations. *Ibis* 2022, 164(4), 1227–1242.
27. Haw C, Lim H, Sodhi N. Space use and habitat selection of house crows in a tropical urban environment: a radio-tracking study. *Raffles Bulletin of Zoology* 2009, 57, 561–568.
28. Hawa A, Azhar B, Top MM, Zubaid A. Home range patterns and sizes of four understorey bird species in secondary peat swamp forests of Selangor, Malaysia. *Malayan Nature Journal* 2019, 71(1).
29. Holcomb KM, Nguyen C, Komar N, Foy BD, Panella NA, Baskett ML, Barker CM. Predicted reduction in transmission from deployment of ivermectin-treated birdfeeders for local control of West Nile virus. *Epidemics* 2023, 44, 100697.
30. Holland AE, Byrne ME, Bryan AL, DeVault TL, Rhodes OE, Beasley JC. Fine-scale assessment of home ranges and activity patterns for resident black vultures (*Coragyps atratus*) and turkey vultures (*Cathartes aura*). *PLoS ONE* 2017, 12(7), e0179819.
31. Houston CS, McLoughlin PD, Mandel JT, Bechard MJ, Stoffel MJ, Barber DR, Bildstein KL. Breeding home ranges of migratory turkey vultures near their northern limit. *Wilson Journal of Ornithology* 2011, 123(3), 472–478.
32. Howe FP, Flake LD. Mourning dove movements during the reproductive season in southeastern Idaho. *Journal of Wildlife Management* 1988, 52(3), 477–480.
33. Jeppesen JL. Territoriality, breeding ranges and relationship between the sexes in pheasant. *Danish Review of Game Biology* 1996.
34. Leif AP. Spatial ecology and habitat selection of breeding male pheasants. *Wildlife Society Bulletin* 2005, 33(1), 130–141.
35. Losito MP, Mirarchi RE. Summertime habitat use and movements of hatching-year mourning doves in northern Alabama. *Journal of Wildlife Management* 1991, 55(1), 137–146.
36. Luukkonen BZ, Klaver RW, Jones OE. Movement of Canada geese in urban and rural areas of Iowa, USA. *Avian Conservation and Ecology* 2022, 17(1).
37. MacLeod CJ, Drew KW, Coleman M. Radio-tracking small farmland passerines: trade-offs in study design. *Notornis* 2011, 58(3&4), 113–123.
38. Møller AP. Advantages and disadvantages of coloniality in the swallow, *Hirundo rustica*. *Animal Behaviour* 1987, 35(3), 819–832.
39. Morganti M, Assandri G, Aguirre JI, Ramirez Á, Caffi M, Pulido F. How residents behave: home range flexibility and dominance over migrants in a Mediterranean passerine. *Animal Behaviour* 2017, 123, 293–304.
40. Morrison JL. Reproductive ecology and habitat associations of Florida's crested caracaras. Thesis, University of Florida; 1997.
41. Naef-Daenzer B. Radiotracking of great and blue tits: new tools to assess territoriality, home-range use and resource distribution. *Ardea* 1994, 82, 335–335.
42. Naef-Daenzer B. Patch time allocation and patch sampling by foraging great and blue tits. *Animal Behaviour* 2000, 59(5), 989–999.
43. Naguib M, Titulaer M, Waas JR, van Oers K, Sprau P, Snijders L. Prior territorial responses and home range size predict territory defense in radio-tagged great tits. *Behavioral Ecology and Sociobiology* 2022, 76(3), 35.
44. Nakamura M, Shindo N. Effects of snow cover on the social and foraging behavior of the great tit *Parus major*. *Ecological Research* 2001, 16(2), 301–308.
45. Peh KS-H, Ong L. A preliminary radio-tracking study of the ranging behaviour of olive-winged bulbul (*Pycnonotus plumosus*) and cream-vented bulbul (*P. simplex*) in a lowland secondary forest in Singapore. *Raffles Bulletin of Zoology* 2002, 50, 251–256.
46. Rappole JH, Tipton AHK, Kane AH. Seasonal effects on control methods for the great-tailed grackle. In: Ninth Great Plains Wildlife Damage Control Workshop Proceedings; Fort Collins, Colorado; 1989. p 120.
47. Ridley MW, Hill DA. Social organization in the pheasant (*Phasianus colchicus*): harem formation, mate selection and the role of mate guarding. *Journal of Zoology* 1987, 211(4), 619–630.
48. Robert RN, David EA. Brood movements of eastern prairie population Canada geese: potential influence of light goose abundance. *Journal of Wildlife Management* 2006, 70(2), 435–442.
49. Rose E, Nagel P, Haag-Wackernagel D. Spatio-temporal use of the urban habitat by feral pigeons (*Columba livia*). *Behavioral Ecology and Sociobiology* 2006, 60(2), 242–254.
50. Rutledge ME, Moorman CE, Washburn BE, Deperno CS. Evaluation of resident Canada goose movements to reduce the risk of goose-aircraft collisions at suburban airports. *Journal of Wildlife Management* 2015, 79(7), 1185–1191.
51. Rutledge ME, Sollmann R, Washburn BE, Moorman CE, DePerno CS. Using novel spatial mark–resight techniques to monitor resident Canada geese in a suburban environment. *Wildlife Research* 2015, 41(5), 447–453.
52. Schmitz RA, Clark WR. Survival of ring-necked pheasant hens during spring in relation to landscape features. *Journal of Wildlife Management* 1999, 63(1), 147–154.
53. Scott AS, Nancy JS, J Edward G. Home ranges, habitat selection and mortality of ring-necked pheasants (*Phasianus colchicus*) in north-central Maryland. *The American Midland Naturalist* 1999, 141(1), 185–197.
54. Humbird SK, Neudorf, DLH. The Effects of Food Supplementation on Extraterritorial Behavior in Female Northern Cardinals. *The Condor: Ornithological Applications* 2008, 110(2), 392–395.
55. Sheng Y, Lu M, Bai J, Xie X, Ma L, Li W, Zhang Z, Ming F, Zhang X, Zhang Z, Xu Z, Han Y, Guan B, Ruan L. Ecological drivers of nesting behavior in a subtropical city: an observational study on spotted doves. *Ecology and Evolution* 2024, 14(7), e11655.
56. Smith JN. Feeding rates, search paths, and surveillance for predators in great-tailed grackle flocks. *Canadian Journal of Zoology* 1977, 55(6), 891–898.
57. Smith WJ, Portugal SJ, Jezierski MT. Use of anthropogenic landscapes in a wild *Columba livia* (rock dove) population. *Ornithology* 2025, 142(1), ukae050.
58. Sol D, Senar JC. Urban pigeon populations: stability, home range, and the effect of removing individuals. *Canadian Journal of Zoology* 1995, 73(6), 1154–1160.
59. van Overveld T, Vardakis M, Arvidsson L, Stolk K, Adriaensen F, Matthysen E. Post-fledging family space use in blue and great tit: similarities and species-specific behaviours. *Journal of Avian Biology* 2017, 48(2), 333–338.
60. Vangestel C, Braeckman BP, Matheve H, Lens L. Constraints on home range behaviour affect nutritional condition in urban house sparrows (*Passer domesticus*). *Biological Journal of the Linnean Society* 2010, 101(1), 41–50.
61. Wang Z, Liu F, Yang W, Zou F. Ecological and behavioral dimensions of coexistence in two understorey babblers in subtropical China. *Current Zoology* 2026, 72(1), 81–94.
62. Whiteside RW, Guthery FS. Ring-necked pheasant movements, home ranges, and habitat use in west Texas. *Journal of Wildlife Management* 1983, 47(4), 1097–1104.
63. Whittaker KA, Marzluff JM. Species-specific survival and relative habitat use in an urban landscape during the post-fledging period. *The Auk* 2009, 126(2), 288–299.

**Supplementary Table S5.** Model selection results (ΔAICc < 7). Continuous variables were log-transformed and standardised prior to model selection for coefficient comparison. All models include random effects of study, site, and species. Models were limited to a maximum of five terms.

| **Model** | **Intercept** | **Context** | **Feather Type** | **Migration** | **Primary Lifestyle** | **Built Area** | **Home Range** | **Primary Lifestyle × Home Range** | **df** | **logLik** | **AICc** | **delta** |
| --- | --- | --- | --- | --- | --- | --- | --- | --- | --- | --- | --- | --- |
| 1 | 3.6176 |  | + |  | + |  | 0.4405 | + | 13 | -75.02 | 178.85 | 0.00 |
| 2 | 3.6203 |  | + |  | + | -0.0327 | 0.4273 | + | 14 | -74.80 | 180.89 | 2.04 |
| 3 | 3.6298 |  | + | + | + |  | 0.4402 | + | 14 | -75.01 | 181.31 | 2.46 |
| 4 | 3.7588 | + | + |  | + |  | 0.4367 | + | 15 | -74.68 | 183.15 | 4.29 |
| 5 | 3.8579 |  |  |  | + |  | 0.3931 | + | 10 | -81.41 | 184.49 | 5.64 |

**Supplementary Table S6.** Variance components from the intercept-only mixed model, partitioning total variance in site-specific feather lead variation coefficients across study, site, species, and residual sources prior to the inclusion of fixed effects.

| **Component** | **Variance** | **SD** | **Variance (%)** |
| --- | --- | --- | --- |
| Study | 0.070 | 0.264 | 19.8 |
| Site | 0.102 | 0.319 | 28.9 |
| Species | 0.098 | 0.313 | 27.8 |
| Residual | 0.083 | 0.288 | 23.5 |

**Supplementary Table S7.** Results of the phylogenetic mixed model, replicating the best-ranked model structure with species random effects replaced by a phylogenetic covariance matrix. Reference categories are terrestrial for primary lifestyle and contour for feather type.

| **Main Effects** | | | | | |
| --- | --- | --- | --- | --- | --- |
| **Predictor** | **Coefficient** | **Std error** | **Z** | **P-value** |  |
| Intercept | 2.927 | 0.275 | 10.639 | <0.001 | *** |
| Ln(Home Range) | 0.207 | 0.043 | 4.756 | <0.001 | *** |
| **Primary Lifestyle** | | | | | |
| Terrestrial (ref.) | - | - | - | - |  |
| Generalist | 0.485 | 0.324 | 1.497 | 0.134 |  |
| Arboreal-Aerial | 1.731 | 0.354 | 4.893 | <0.001 | *** |
| **Feather Type** | | | | | |
| Contour (ref.) | - | - | - | - |  |
| Wing | 0.249 | 0.124 | 2.002 | 0.045 | * |
| Tail | 0.244 | 0.128 | 1.913 | 0.056 | . |
| Mixed | 0.628 | 0.175 | 3.591 | <0.001 | *** |
| **Interactions** | | | | | |
| Ln(Home Range) × Terrestrial (ref.) | - | - | - | - |  |
| Ln(Home Range) × Generalist | -0.060 | 0.085 | -0.706 | 0.480 |  |
| Ln(Home Range) × Arboreal-Aerial | -0.464 | 0.111 | -4.165 | <0.001 | *** |

| **Random Effects** | | | |
| --- | --- | --- | --- |
| **Component** | **Variance** | **SD** | **Variance (%)** |
| Study | 0.058 | 0.241 | 41.7% |
| Site | 0.060 | 0.244 | 42.6% |
| Phylogeny | 0.014 | 0.120 | 10.3% |
| Species | <0.001 | <0.001 | <0.1% |
| Residual | 0.087 | 0.295 | 5.4% |

**Supplementary Table S8.** Fixed effects in the top-ranked linear mixed model, with Kenward-Roger degrees of freedom. F-values test the overall significance of each term across all its levels. Numerator degrees of freedom (NumDF) indicate the number of parameters tested per term. Denominator degrees of freedom (DenDF) indicate the effective sample size available to estimate each effect after accounting for the random effects structure.

| **Predictor** | **Sum Sq** | **Mean Sq** | **NumDF** | **DenDF** | **F value** | **P-value** |
| --- | --- | --- | --- | --- | --- | --- |
| Feather Type | 0.992 | 0.331 | 3 | 56.3 | 3.935 | 0.013 |
| Primary Lifestyle | 1.984 | 0.992 | 2 | 26.3 | 11.849 | <0.001 |
| Ln(Home Range) | 0.075 | 0.075 | 1 | 27.7 | 0.901 | 0.351 |
| Primary Lifestyle × Ln(Home Range) | 1.313 | 0.657 | 2 | 31.6 | 7.823 | 0.002 |

**Supplementary Table S9.** Post hoc contrasts from the best-ranked linear mixed model, with Kenward-Roger degrees of freedom. (A) Estimated marginal means for feather type with 95% confidence intervals. (B) Pairwise contrasts between feather types, Tukey adjusted. (C) Home range slope estimates by primary lifestyle group, tested against zero with Holm adjustment. (D) Pairwise comparisons of home range slopes between primary lifestyle groups, Tukey adjusted. All values are on the log scale.

**(A) Feather type marginal means**

| **Feather Type** | **Marginal mean** | **SE** | **df** | **95% CI** |
| --- | --- | --- | --- | --- |
| Contour | 3.734 | 0.121 | 42.1 | 3.490, 3.978 |
| Tail | 3.959 | 0.122 | 38.3 | 3.712, 4.206 |
| Wing | 3.967 | 0.122 | 45.1 | 3.721, 4.212 |
| Mixed | 4.360 | 0.163 | 40.8 | 4.031, 4.689 |

**(B) Feather type pairwise contrasts**

| **Contrast** | **Estimate** | **SE** | **df** | **t** | **P-value** |  |
| --- | --- | --- | --- | --- | --- | --- |
| Contour − Mixed | −0.626 | 0.185 | 40.7 | −3.391 | 0.008 | ** |
| Contour − Tail | −0.225 | 0.136 | 67.9 | −1.651 | 0.358 |  |
| Contour − Wing | −0.233 | 0.134 | 70.1 | −1.742 | 0.310 |  |
| Mixed − Tail | 0.401 | 0.185 | 42.7 | 2.161 | 0.151 |  |
| Mixed − Wing | 0.393 | 0.185 | 42.4 | 2.123 | 0.162 |  |
| Tail − Wing | −0.008 | 0.119 | 59.6 | −0.064 | 1.000 |  |

**(C) Home range slopes by primary lifestyle**

| **Primary Lifestyle** | **Slope** | **SE** | **df** | **t** | **P-value** | **95% CI** |  |
| --- | --- | --- | --- | --- | --- | --- | --- |
| Terrestrial | 0.222 | 0.044 | 21.8 | 5.043 | <0.001 | 0.131, 0.313 | *** |
| Generalist | 0.145 | 0.077 | 29.9 | 1.882 | 0.070 | −0.012, 0.302 | . |
| Arboreal-Aerial | −0.237 | 0.104 | 27.4 | −2.271 | 0.062 | −0.451, −0.023 | . |

**(D) Pairwise slope comparisons**

| **Contrast** | **Estimate** | **SE** | **df** | **t** | **P-value** |  |
| --- | --- | --- | --- | --- | --- | --- |
| Terrestrial − Generalist | 0.077 | 0.088 | 29.5 | 0.873 | 0.661 |  |
| Terrestrial − Arboreal-Aerial | 0.459 | 0.116 | 32.3 | 3.961 | 0.001 | ** |
| Generalist − Arboreal-Aerial | 0.382 | 0.128 | 31.8 | 2.985 | 0.015 | * |

**Supplementary Table S10.** Bootstrap retention scores from a grouped lasso analysis of site-specific variability in feather lead concentrations. All continuous predictors were log-transformed and standardised prior to analysis. A grouped lasso penalty (*grLasso*) was applied via 10-fold cross-validation to identify the regularisation parameter (λ), with the most conservative model within one standard error of minimum cross-validation error used as the selection threshold. Retention scores reflect the percentage of 1,000 bootstrap resamples in which each term group was retained at this selection threshold.

| **Term** | **Retention (%)** |
| --- | --- |
| Home Range × Primary Lifestyle | 99.7 |
| Feather Type | 99.6 |
| Home Range × Trophic Level | 96.9 |
| Home Range | 91.2 |
| Body Mass × Context | 79.1 |
| Trophic Level × Built Area | 74.9 |
| Body Mass × Feather Type | 68.4 |
| Primary Lifestyle | 62.7 |
| Body Mass | 57.7 |
| Feather Type × Built Area | 55.4 |
| Migration × Built Area | 45.7 |
| Feather Washed | 45.0 |
| Body Mass × Primary Lifestyle | 42.8 |
| Primary Lifestyle × Built Area | 32.8 |
| Trophic Level | 30.0 |
| Home Range × Migration | 27.4 |
| Home Range × Feather Type | 22.1 |
| Built Area × Feather Washed | 22.0 |
| Body Mass × Trophic Level | 21.1 |
| Body Mass × Migration | 15.5 |
| Home Range × Feather Washed | 9.2 |
| Body Mass × Feather Washed | 7.7 |
| Context | 6.7 |
| Home Range × Context | 6.7 |
| Built Area × Context | 4.8 |
| Built Area | 4.6 |
| Migration | 1.0 |

**Supplementary Table S11.** Results of the best-ranked model from re-running model selection on the Cook's distance sensitivity analysis dataset, with the top 5% most influential observations removed. Coefficients are based on log transformed variation coefficients and home range sizes, with Kenward-Roger degrees of freedom. Reference categories are Terrestrial for Primary Lifestyle and Contour for Feather Type.

| **Main Effects** | | | | | | |
| --- | --- | --- | --- | --- | --- | --- |
| **Predictor** | **Coefficient** | **Std error** | **df** | **t-value** | **P-value** |  |
| Intercept | 2.660 | 0.205 | 12.3 | 12.965 | <0.001 | *** |
| Ln(Home Range) | 0.243 | 0.035 | 18.4 | 6.887 | <0.001 | *** |
| **Primary Lifestyle** | | | | | | |
| Terrestrial (ref.) | - | - | - | - | - |  |
| Generalist | 0.609 | 0.273 | 20.1 | 2.230 | 0.037 | * |
| Arboreal-Aerial | 2.112 | 0.313 | 29.4 | 6.742 | <0.001 | *** |
| **Feather Type** | | | | | | |
| Contour (ref.) | - | - | - | - | - |  |
| Wing | 0.336 | 0.122 | 70.0 | 2.747 | 0.008 | ** |
| Tail | 0.309 | 0.124 | 67.5 | 2.495 | 0.015 | * |
| Mixed | 0.826 | 0.170 | 40.5 | 4.855 | <0.001 | *** |
| **Interactions** | | | | | | |
| Ln(Home Range) × Terrestrial (ref.) | - | - | - | - | - |  |
| Ln(Home Range) × Generalist | -0.086 | 0.073 | 29.4 | -1.186 | 0.245 |  |
| Ln(Home Range) × Arboreal-Aerial | -0.673 | 0.122 | 40.2 | -5.493 | <0.001 | *** |

| **Random Effects** | | | |
| --- | --- | --- | --- |
| **Component** | **Variance** | **SD** | **Variance (%)** |
| Study | 0.054 | 0.233 | 33.5 |
| Site | 0.033 | 0.181 | 20.2 |
| Species | <0.001 | <0.001 | <0.1 |
| Residual | 0.075 | 0.274 | 46.3 |

**Supplementary Table S12.** Results of the best-ranked model from re-running model selection on the urbanised landscape subset (>20% built cover; n = 113). Coefficients are based on log transformed variation coefficients and home range sizes, with Kenward-Roger degrees of freedom. Reference categories are Terrestrial for Primary Lifestyle and Contour for Feather Type.

| **Main Effects** | | | | | | |
| --- | --- | --- | --- | --- | --- | --- |
| **Predictor** | **Coefficient** | **Std error** | **df** | **t-value** | **P-value** |  |
| Intercept | 2.815 | 0.276 | 13.1 | 10.202 | <0.001 | *** |
| Ln(Home Range) | 0.175 | 0.049 | 13.7 | 3.585 | 0.003 | ** |
| **Primary Lifestyle** | | | | | | |
| Terrestrial (ref.) | - | - | - | - | - |  |
| Generalist | 0.272 | 0.215 | 10.4 | 1.265 | 0.233 |  |
| Arboreal-Aerial | 0.584 | 0.235 | 10.6 | 2.487 | 0.031 | * |
| **Feather Type** | | | | | | |
| Contour (ref.) | - | - | - | - | - |  |
| Wing | 0.449 | 0.154 | 41.6 | 2.914 | 0.006 | ** |
| Tail | 0.528 | 0.154 | 40.4 | 3.424 | 0.001 | ** |
| Mixed | 0.939 | 0.212 | 27.5 | 4.435 | <0.001 | *** |

| **Random Effects** | | | |
| --- | --- | --- | --- |
| **Component** | **Variance** | **SD** | **Variance (%)** |
| Study | 0.035 | 0.188 | 15.4 |
| Site | 0.061 | 0.246 | 26.4 |
| Species | 0.036 | 0.189 | 15.5 |
| Residual | 0.098 | 0.313 | 42.7 |
